# Brain–heart interaction after acute ischemic stroke

**DOI:** 10.1186/s13054-020-02885-8

**Published:** 2020-04-21

**Authors:** Denise Battaglini, Chiara Robba, Adriana Lopes da Silva, Cynthia dos Santos Samary, Pedro Leme Silva, Felipe Dal Pizzol, Paolo Pelosi, Patricia Rieken Macedo Rocco

**Affiliations:** 1 Anesthesia and Intensive Care, San Martino Policlinico Hospital, IRCCS for Oncology and Neurosciences, Genoa, Italy; 2 Department of Surgical Sciences and Integrated Diagnostics, University of Genoa, Genoa, Italy; 3 Laboratory of Pulmonary Investigation, Carlos Chagas Filho Institute of Biophysics, Federal University of Rio de Janeiro, Rio de Janeiro, Brazil; 4 Department of Physiotherapy, Faculty of Medicine, Federal University of Rio de Janeiro, Rio de Janeiro, Brazil; 5 Unidade Acadêmica de Ciências da Saude, Universidade do Extremo Sul Catarinense (UNESC), Criciúma, Santa Catarina Brazil; 6 Rio de Janeiro Network on Neuroinflammation, Carlos Chagas Filho Foundation for Supporting Research in the State of Rio de Janeiro (FAPERJ), Rio de Janeiro, Brazil

**Keywords:** Acute ischemic stroke, Heart, Arrhythmia, Cerebrovascular, Cardiovascular, Neuroinflammation

## Abstract

Early detection of cardiovascular dysfunctions directly caused by acute ischemic stroke (AIS) has become paramount. Researchers now generally agree on the existence of a bidirectional interaction between the brain and the heart. In support of this theory, AIS patients are extremely vulnerable to severe cardiac complications. Sympathetic hyperactivity, hypothalamic–pituitary–adrenal axis, the immune and inflammatory responses, and gut dysbiosis have been identified as the main pathological mechanisms involved in brain–heart axis dysregulation after AIS. Moreover, evidence has confirmed that the main causes of mortality after AIS include heart attack, congestive heart failure, hemodynamic instability, left ventricular systolic dysfunction, diastolic dysfunction, arrhythmias, electrocardiographic anomalies, and cardiac arrest, all of which are more or less associated with poor outcomes and death. Therefore, intensive care unit admission with continuous hemodynamic monitoring has been proposed as the standard of care for AIS patients at high risk for developing cardiovascular complications. Recent trials have also investigated possible therapies to prevent secondary cardiovascular accidents after AIS. Labetalol, nicardipine, and nitroprusside have been recommended for the control of hypertension during AIS, while beta blockers have been suggested both for preventing chronic remodeling and for treating arrhythmias. Additionally, electrolytic imbalances should be considered, and abnormal rhythms must be treated. Nevertheless, therapeutic targets remain challenging, and further investigations might be essential to complete this complex multi-disciplinary puzzle. This review aims to highlight the pathophysiological mechanisms implicated in the interaction between the brain and the heart and their clinical consequences in AIS patients, as well as to provide specific recommendations for cardiovascular management after AIS.

## Introduction

Cardiovascular disease is regarded as the main predisposing risk factor for acute ischemic stroke (AIS) [1]. Cardiac dysfunction can both worsen the pre-existing cerebral damage and cause a new brain injury. AIS incidence is doubled in patients with coronary heart disease and increased fivefold for those with atrial fibrillation [1]. Additionally, since brain damage can modify the autonomic and neurohormonal pathways involved in the control of heart function, patients affected by stroke are extremely vulnerable to severe cardiac adverse events [2]. In particular, AIS can contribute to impaired cerebral autoregulation, thus making cerebral blood flow directly dependent on cardiac function [3]. In this context, the concept of a two-way interaction between the brain and heart has been proposed [4, 5]. The aim of this systematic review is to highlight the pathophysiological mechanisms implicated in the interaction between the brain and the heart and their clinical consequences in AIS patients, as well as to provide specific recommendations for cardiovascular management after AIS.

## Epidemiology of cardiac dysfunction in ischemic stroke

The incidence of cardiovascular complications after AIS ranges from 3% for myocardial infarction to > 50% for asymptomatic coronary stenosis [6]. The most serious complications after AIS have been identified in the acute phase [5], and the risk of developing cardiac complications is proportional to the severity of AIS [7]. Likewise, impaired cardiac function after AIS increases the risk of worse neurologic outcomes and 90-day disability [8]. Following AIS, 24% of patients develop autonomic dysfunction [9], 28% show impairment of left ventricular ejection fraction, and 13–29% develop systolic dysfunction [5]. Electrocardiographic abnormalities are observed in 60–85% of AIS patients within the first 24 h [10, 11] (Table S1, Additional file 1).

## Pathophysiological mechanisms involved in stroke-heart interaction

The main mechanisms involved in the so-called stroke-heart crosstalk include the hypothalamic–pituitary–adrenal axis (HPA) [12], the immune and inflammatory responses [13], and the gut dysbiosis [14], as well as the risk factors (age, sex, race, hypertension, smoking, diet, and physical inactivity) primarily involved in the pathogenesis of AIS [15]. Regardless of the mechanisms, cardiac complications increase the risk of developing AIS, and vice-versa; AIS causes dysautonomia and hyperinflammation, thus predisposing patients to heart dysfunction [16].

### Dysregulated autonomic activity after AIS

#### Central autonomic network: from the brain to the heart

**Fig. 1:**
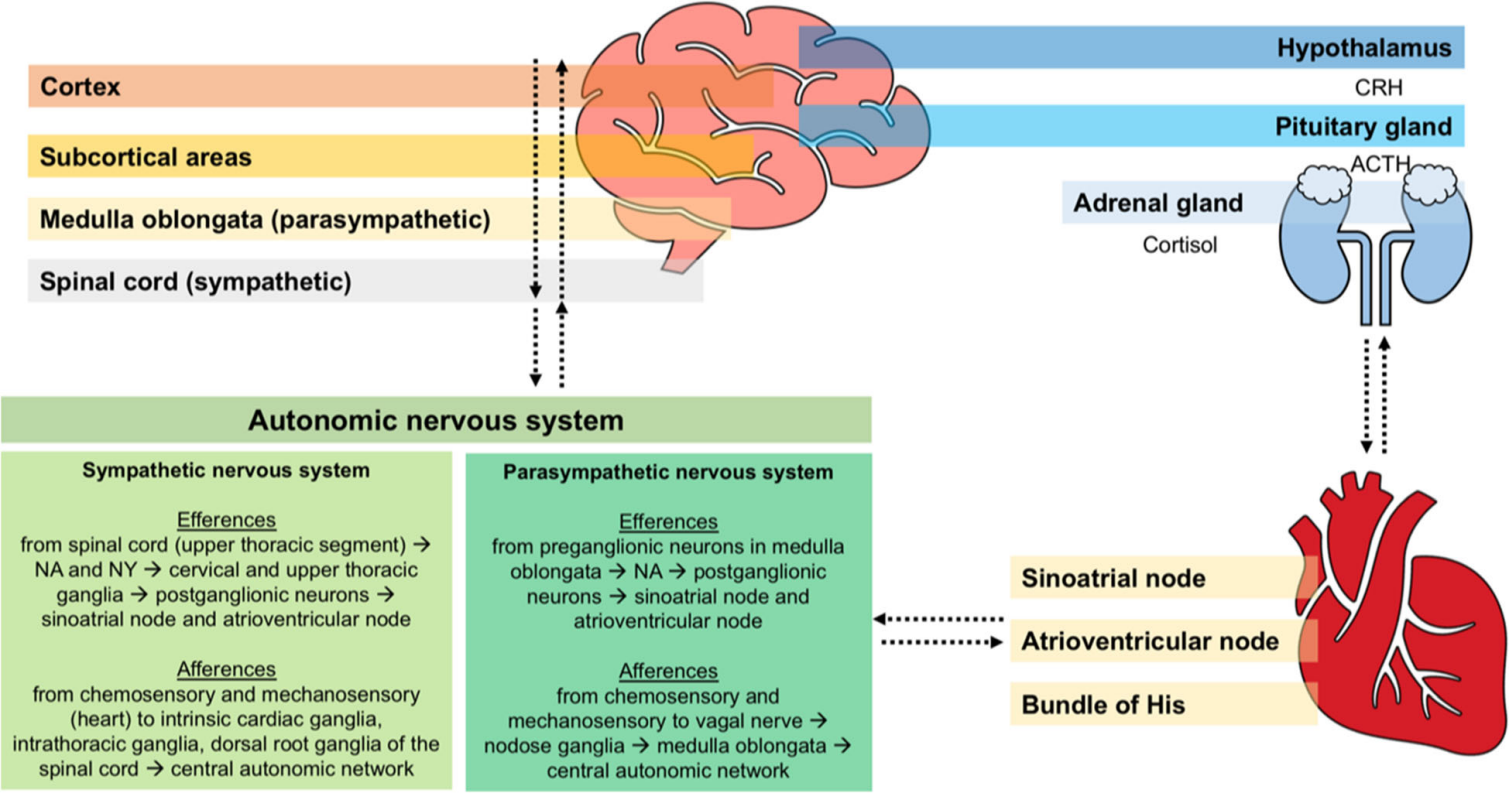
Neural connections: from the brain to the heart and from the heart to the brain. Sympathetic system efferences from the spinal cord (NA and NY) to the cervical and upper thoracic ganglia, thus to sinus atrial node and atrial ventricular node. Afferences from the chemosensory and mechanosensory neurons in the heart to the intrathoracic and dorsal root ganglia of the spinal cord, to central autonomic network. Parasympathetic system efferences from the medulla oblongata (noradrenaline) to the sinus atrial node and atrial ventricular node. Afferences from the chemosensory and mechanosensory neurons to the vagal nerve, nodose ganglia, medulla oblongata, and central autonomic network. Efferences from the hypothalamus (CRH) to the pituitary gland (ACTH) to the adrenal gland (cortisol). NA, noradrenaline; NY, neuropeptide Y; CRH, corticotropin-releasing hormone; ACTH, adrenocorticotropic hormone

**Fig. 2:**
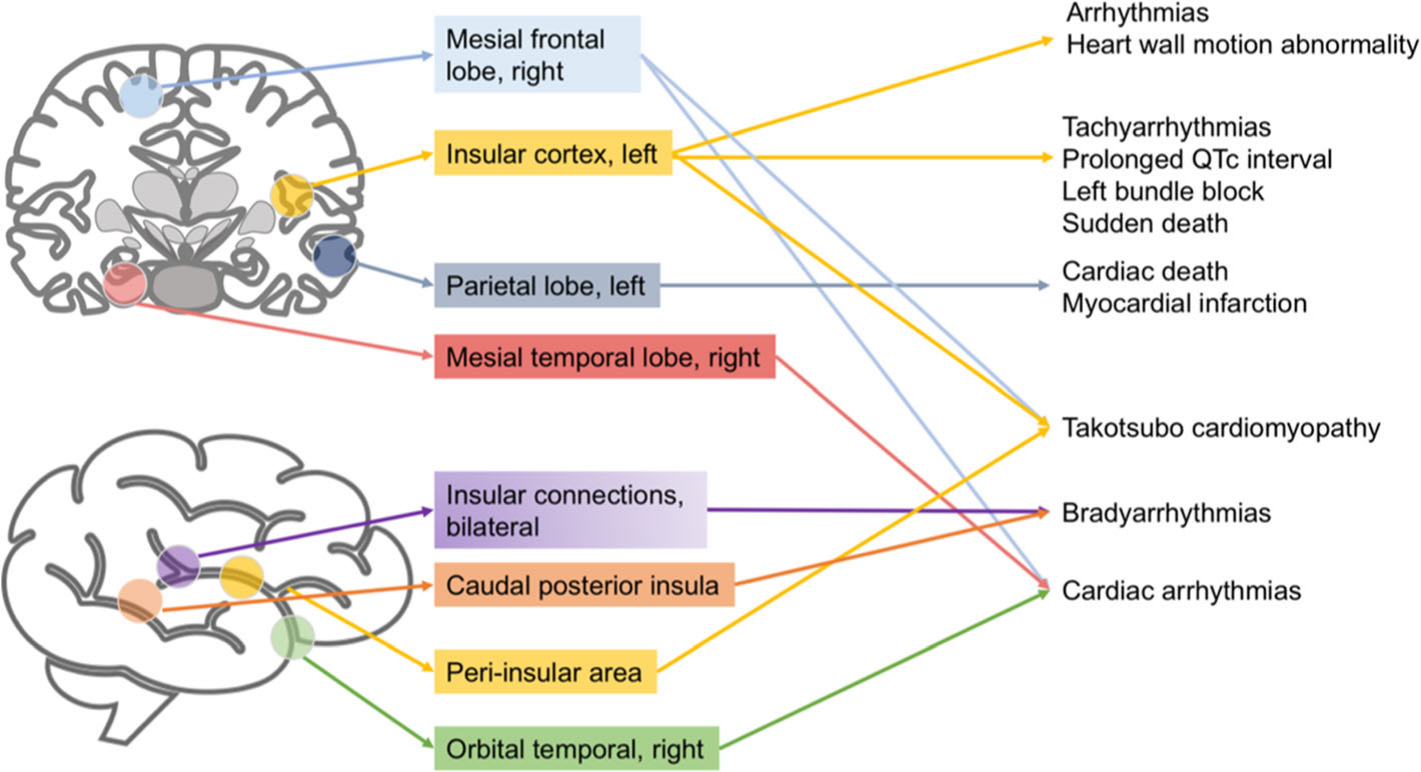
Activation of different brain areas during stroke followed by specific cardiovascular complications. Depending on the extent of subsequent brain damage, stroke triggers different central regulatory regions, thus activating corresponding pathways that depend on the injured area. Therefore, post-stroke cardiac dysfunctions may be referred to specific brain areas. Right-sided stroke is usually associated with more cardiac complications than left-sided stroke. QTc, corrected QT interval

Central pathways regulating autonomic responses from the brain to the heart involve structures implicated in physiological, pathological, and emotional responses [17] (Fig. 1). When brain damage occurs, each central regulatory region triggers different pathways that depend on the injured area involved and on the extent of injury (Fig. 2). Stimulation of the orbital surface of the frontal lobe and cingulate gyrus, for instance, alters blood pressure and heart rate control; ischemic lesions of the insular cortex affect blood pressure control and trigger serious cardiac complications, such as arrhythmias and autonomic dysfunction [9, 18]. Moreover, left hemisphere brain infarction is associated with a greater risk of adverse cardiac outcomes and increased long-term mortality [19].

#### Enhanced sympathetic activity

**Fig. 3:**
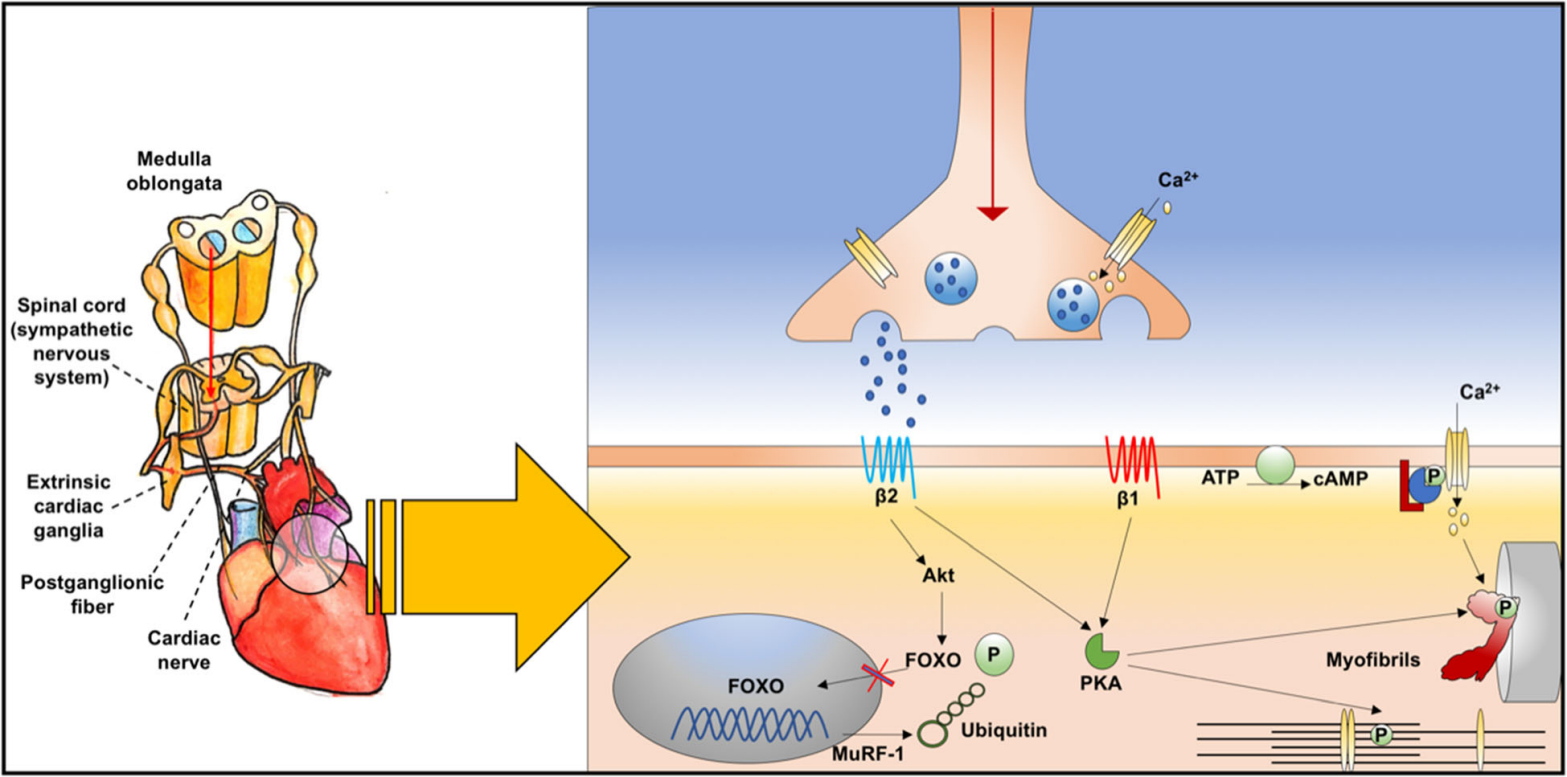
Brain–heart sympathetic pathway at the molecular level. The “fight or flight” response of catecholaminergic storm, followed by hypothalamic–pituitary–adrenal axis and autonomic activation, is represented at the molecular level. Synaptic connection through neurons and myocytes is represented. Noradrenaline activates β1 receptors, which in turn activates cyclic adenosine monophosphate–protein kinase A (cAMP–PKA) signaling, with consequent release of Ca^2+^ from the sarcoplasmic reticulum for cell contraction. At the same time, noradrenaline activates β2 receptors, which, acting through the protein kinase B (Akt)-FOXO pathway, decrease protein degradation by ubiquitin, thus regulating cardiomyocyte proteostatic equilibrium and cardiac mass maintenance with *muscle ring finger-1* (MuRF-1), which is upregulated in the deficient heart. FOXO, forkhead box O; Akt, protein kinase B; PKA, protein kinase A; cAMP, cyclic adenosine monophosphate, ATP, adenosine triphosphate; MuRF-1, muscle ring finger-1. Modified from "Martini FH. Fundamentals of Anatomy and Physiology. 8th ed. 2006. Chapter 20"

Sympathetic connections between the central nucleus and the heart are mediated by sympathetic pre-ganglionic neurons in the upper thoracic segment of the spinal cord, synaptic connections in the cervical and upper thoracic ganglia, and sympathetic post-ganglionic neurons [17]. The “fight or flight” response of catecholaminergic storm after HPA axis and autonomic activation is followed at the molecular level by activation of forkhead box O (*FOXO*) genes. *FOXO* genes have been recently identified as a potential new molecular target for cardiac dysfunction and are associated with increased risk of myocardial infarction [20]. Noradrenaline activates β1 receptors; this, in turn, activates cyclic adenosine monophosphate–protein kinase A signaling, with a consequent release of calcium from the sarcoplasmic reticulum for cell contraction. At the same time, noradrenaline activates β2 receptors, which, acting through the protein kinase B (Akt)-*FOXO* pathway, decrease protein degradation by ubiquitin, thus regulating cardiomyocyte proteostatic equilibrium and cardiac mass maintenance with muscle ring finger-1, a class of proteins that is upregulated in a deficient heart [20]. The consequences of this catecholamine surge are cardiomyocyte necrosis, hypertrophy, fibrosis, and cardiac arrhythmias [20] (Fig. 3).

#### Enhanced parasympathetic activity

Parasympathetic connections include noradrenergic pre-ganglionic neurons in the medulla oblongata, nucleus ambiguus, vagus nerve, and reticular formation [17]. These nuclei connect with the epicardial ganglionated plexus, communicating through post-ganglionic fibers that release acetylcholine and vasoactive intestinal peptide [17]. By binding type 2 muscarinic receptors, acetylcholine reduces intracellular cyclic adenosine monophosphate levels, thus slowing the speed of depolarization. Activation of this pathway results in lengthening of atrioventricular conduction time and reduces ventricular contractility [17] (Fig. 1).

#### Reflex activation of cardiac autonomic nerves: from the heart to the brain

Baroreceptor and chemoreceptor afferent neurons reach the solitary nucleus, and signals are transmitted to cardiac neurons (via glutamatergic neurons), to the caudal ventrolateral medulla (via GABAergic neurons), or to the rostral ventrolateral medulla. After input integration, the central autonomic network re-transmits signals to the heart via the parasympathetic and the sympathetic systems [17] (Fig. 1).

#### Catecholamine release

Adrenocorticotropic hormone activates the adrenal gland to release cortisol, followed by catecholamines, which, by binding β1 adrenoreceptor, modifies intracellular calcium levels, induces oxidative stress, reduces adenosine triphosphate synthesis, and leads to osmotic swelling, which causes myocardial cell death [21].

### The local and systemic inflammatory response to ischemic stroke

**Fig. 4:**
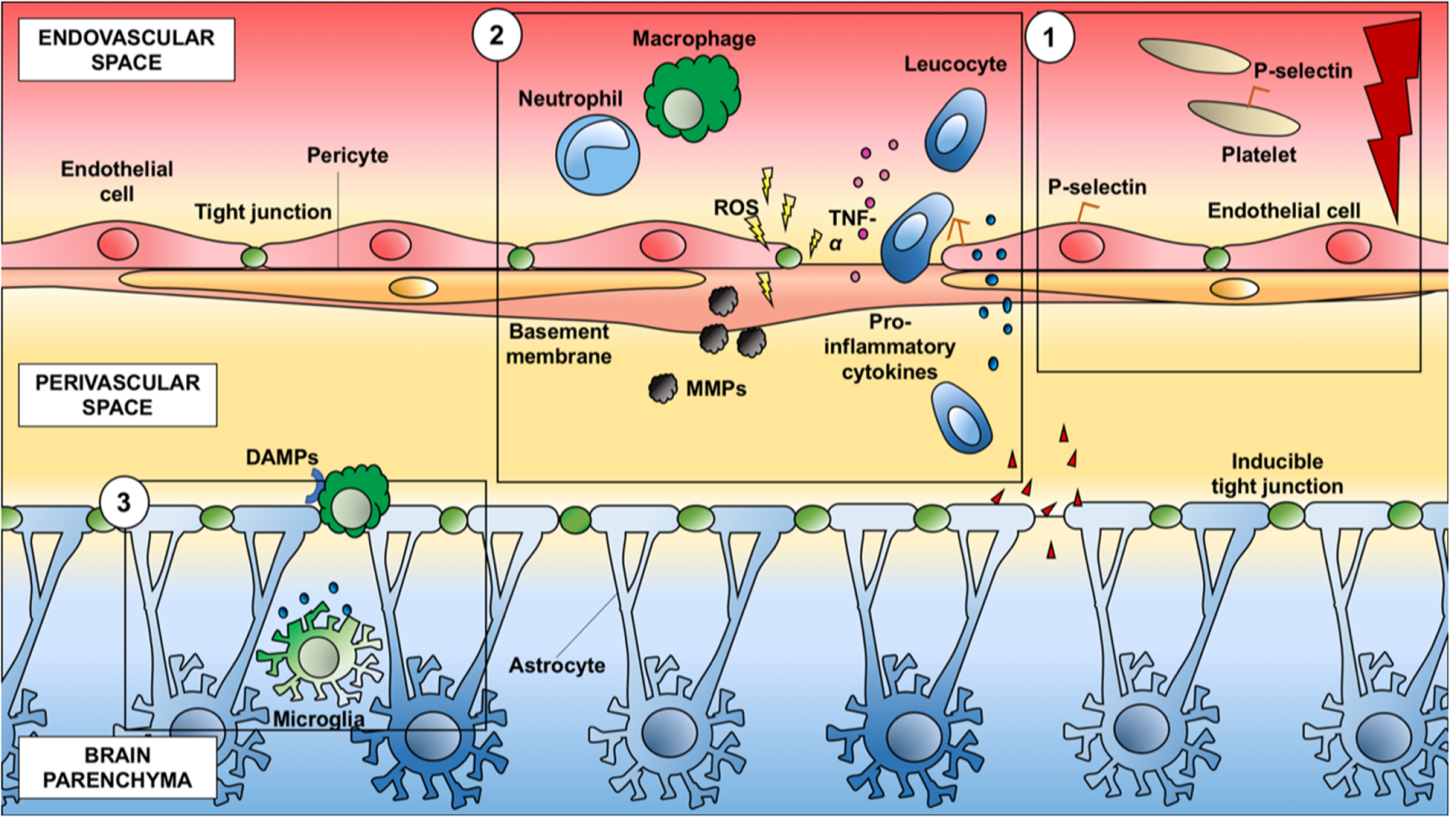
Local inflammatory response after stroke. The local inflammatory process starts with the activation of pro-inflammatory and pro-coagulative cascades into the intravascular space. The blood–brain barrier disruption allows the infiltration of peripheral macrophages and neutrophils into the ischemic lesion. This leads to an enhanced local inflammatory response in the brain parenchyma. Other peripheral immune cells are thus recalled into the ischemic brain and cerebral microcirculation, subsequently crossing the damaged blood–brain barrier and passing into the systemic circulation. ROS, reactive oxygen species; TNF-α, tumor necrosis factor alpha; MMPs, matrix metalloproteinases; DAMPs, danger-associated molecular patterns

The immune inflammatory response plays a prominent role immediately after AIS, and is strongly associated with ischemic stroke progression [22]. During the early phase of AIS, elements of both innate and adaptive immunity are involved in local and systemic inflammatory cascades [22]. The local inflammatory process starts with the activation of pro-inflammatory and pro-coagulative cascades into the endovascular space after vessel occlusion, within a few minutes of ischemia. P-selectin, a cell adhesion molecule, is present in platelets and endothelial cells (Fig. 4, phase 1). Macrovesicles are subsequently recruited and bind to P-selectin, causing platelet homing and thrombus formation. Blood–brain barrier (BBB) disruption allows infiltration of peripheral macrophages and neutrophils into the ischemic lesion, passing through the perivascular space [23] (Fig. 4, phase 2). This leads to an enhanced local inflammatory response in the brain parenchyma, which includes cytokines and chemokines, microgliosis, astrogliosis, and endothelial cell activation. Resident microglia and macrophages are converted to the M1-phenotype and attracted into the lesion, passing through the damaged BBB. Endothelial cells are also damaged by increasing oxidative stress, and by metalloproteinases produced by neutrophils [24], thus inducing BBB injury [23]. Thus, adenosine triphosphate levels increase, producing hyperpolarization of glial cells and enhancing inflammation. At this stage, pro-inflammatory cytokines are activated. Other peripheral immune cells are thus recalled into the ischemic brain and cerebral microcirculation, subsequently crossing the damaged BBB and passing into the systemic circulation [23] (Fig. 4, phase 2). The post-ischemic brain releases danger-associated molecular patterns capable of activating Toll-like receptors and scavenger receptors that are expressed on perivascular macrophages, microglia, and endothelial brain cells. These patterns are responsible for antigen presentation by dendritic cells [23] (Fig. 4, phase 3). The local inflammatory response is extended into the systemic circulation, yielding possible secondary cardiac damage. An in vitro test in rat heart myocytes demonstrated that, after 90 min of oxygen-glucose deprivation, the cells were exposed to ischemic–reperfusion injury. Likewise, an in vivo study found expression of markers of necrosis, apoptosis, and autophagy in these cells [25]. Neural and cardiac myocyte cell death has been recently explored in other experimental settings, suggesting a potential correlation between post-AIS inflammation and cardiac dysfunction [26]. Chronic inflammation and apoptosis were found in the cerebellum and heart of non-human primates 6 months after transient global ischemia induction [27].

### Gut microbiome dysbiosis after AIS

**Fig. 5:**
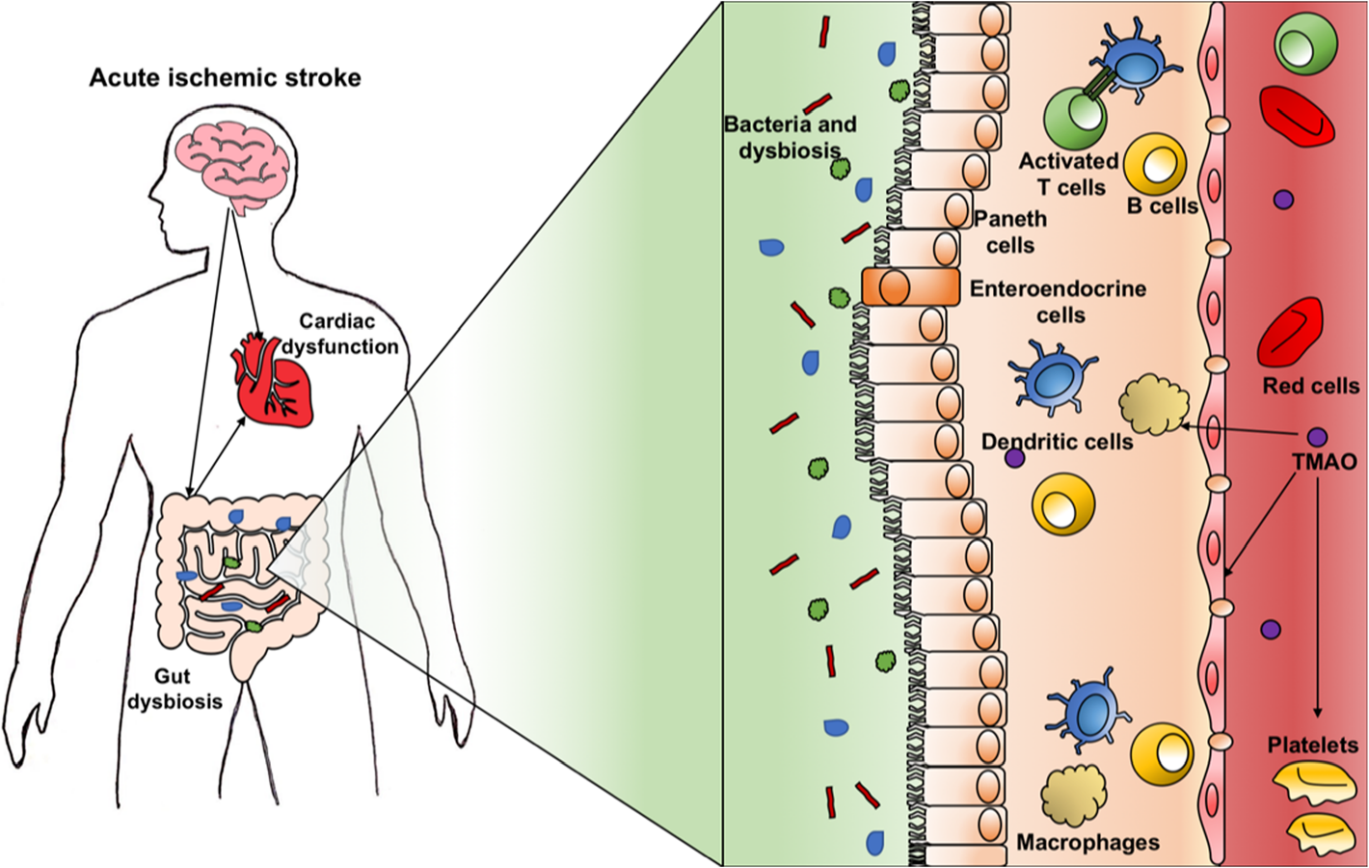
Gut dysbiosis and cardiac dysfunction. Gut dysbiosis causes increased gut–blood barrier permeability and pathogen translocation, with possible atherosclerosis and thrombosis. Gut pathogens contribute to enhance the inflammatory response through platelet hyperactivation and thrombosis, mediated by the conversion of choline and l-carnitine into trimethylamine N-oxide (TMAO). TMAO induces platelet hyperactivity and foam cell formation, alters bile and sterol metabolism, and activates macrophages, dendritic cells, and platelets. TMAO, trimethylamine N-oxide

After a brain insult, up to 50% of AIS patients develop gastrointestinal complications such as dysphagia, gastrointestinal hemorrhage, and intestinal paralysis, which can have a major impact on neurological outcomes and mortality [28]. The gut–blood barrier is responsible for the absorption of water and nutrients while preventing passage of toxins and pathogens into the blood [14]. Several injurious mechanisms can alter intestinal permeability and cause disruption of the gut–blood barrier, thus increasing its permeability and dysregulating the gut microbiota [28]. The greater the alteration in intestinal permeability, the greater the severity of stroke [14]. After AIS, the gut commensal flora is imbalanced in favor of pathogens, which may influence post-AIS outcomes [29]. Likewise, the amount of pathogens in the bowel after AIS has been associated with the severity of inflammatory response [29]. Gut pathogens contribute to the inflammatory response through platelet hyperactivation and thrombosis, mediated by conversion of choline and l-carnitine into trimethylamine-N-oxide (TMAO). TMAO induces platelet hyperactivity and foam cell formation, alters bile and sterol metabolism, increases nuclear factor kappa-B, and reduces nitric oxide. It also enhances the inflammatory response by acting on dendritic cells, macrophages, and platelets. These mechanisms are responsible for heart failure, cardiac remodeling, myocardial infarction, thrombosis, and atherosclerosis [30]. Some clinical trials have identified TMAO as a marker and predictor of cardiovascular disease [29]. Its levels have been associated with impaired cardiac function, heart attack, and heart failure [31] (Fig. 5).

### AIS and cardiovascular risk factors

Identification of risk factors for AIS is complicated by the fact that both cardiac dysfunction and AIS share the same factors. Hypertension may contribute to atherosclerotic disease, which is prominent in the pathophysiology of AIS [15]. Moreover, it may contribute to cardiovascular events, leading to increased difficulty when interpreting whether AIS or pre-existing risk factors are causative for post-AIS cardiac dysfunction [32]. Many scores have been identified for detecting patients who can be at risk for AIS or cardiovascular disease, since they exhibit similar risk factors. These include the Framingham Risk Score and the Pooled Cohort Equations [15, 32]. One of the main causes of AIS may be a cerebral vessel embolism, originating from an atherosclerotic ulcerated plaque, frequently located in the internal carotid artery [33]. It is not clear whether AIS, with its inflammatory consequences, is the main causative factor for post-AIS cardiac dysfunction or if a pre-AIS cardiac predisposition affects post-stroke cardiovascular events. Concerning atherosclerotic plaques, minimal vessel disease in the small arteries and arterioles of the brain can promote lacunar AIS, cerebral hemorrhage, and leukoaraiosis. The exposure of the small vessels to high pressure could further induce lipohyalinosis followed by sub-cortical infarct [33].

## Clinical implications

Cardiac issues that may be implicated in AIS include atrial fibrillation, patent foramen ovale, a hypokinetic segment with mural thrombus, infective endocarditis, and hematological disorders (e.g., polycythemia vera) [33]. Based on experimental and clinical evidence, patients affected by AIS are extremely vulnerable to severe cardiac adverse events [6], since they frequently already suffer from cardiac dysfunction [33]. Studies suggest that heart failure after AIS occurs in up to 17% of cases, even in patients without a preexisting cardiac disease [34]. Cardiac death and serious cardiac events are reported in 4% and 19% of patients, respectively, within the first 3 months after an AIS [35]. Cardiac arrhythmias [18], Takotsubo cardiomyopathy [7], myocardial infarction [36], autonomic dysfunction [37], and paroxysmal hypertension [38] are the most common clinical features (Table S1, Additional file 1). Additionally, the localization of the stroke area involved is a fundamental predictor of possible cardiac consequences (Fig. 2). In this line, left parietal lobe infarction is an independent risk factor for myocardial infarction [39]. Although the insular cortex has a major role in the control of the autonomic system, stroke occurrence in this area is not very common [40]. However, when right insular damage occurs, the risk of tachyarrhythmias, prolonged QTc interval, and left bundle block is increased [41]. Right-sided stroke might be more likely associated with parasympathetic modulation than left-sided stroke [38, 42]; a similar association has been described with cardiac arrhythmias [43]. Takotsubo cardiomyopathy has also been related to specific ischemic areas, such as the frontal cortex and sub-cortex, insular and peri-insular areas, and caudal posterior insula [44].

### Acute and chronic cardiac dysfunction pre-AIS

Atrial fibrillation and flutter cause blood stagnation in the left atrium, thus predisposing to thrombosis and cerebral embolism [45]. Valvular atrial fibrillation increases the risk of AIS 17-fold, while non-valvular atrial fibrillation increases the risk 5-fold [45]. No distinction has been made in terms of rate of risk for AIS among paroxysmal, permanent, or persistent atrial fibrillation, thus making a more aggressive monitoring strategy difficult, especially in patients with an initial diagnosis of an arrhythmia other than atrial fibrillation [45]. Systolic blood pressure higher than 140 mmHg is responsible for 1.5 million AIS-related deaths [46]. Moreover, high blood pressure is linked to increased risk of atrial fibrillation, which is itself a well-known risk factor for AIS [47]. Chronic heart failure is also considered an important risk factor for AIS, as it is associated with thrombus formation, left ventricular hypokinesia, increased blood viscosity, reduced left ventricular ejection fraction, endothelial dysfunction, systolic or diastolic heart dysfunction, and small vessel occlusion, with consequent poor outcome and higher mortality [48].

### Acute and chronic cardiac dysfunction after AIS

#### Acute cardiac dysfunction after AIS

Both ischemic and arrhythmic electrocardiographic changes are common after AIS within the first 24 h [18]. Additionally, acute myocardial infarction or myocardial infarction-like abnormalities are very common after AIS [36] and are associated with an increased risk of unfavorable functional neurological outcomes and major cardiovascular events [38].

##### Cardiac arrhythmias

Electrocardiographic changes occur in 60–90% of AIS patients [10, 11]. Common features include T wave inversion (35%), ST depression (33%), prolonged QTc interval (29%), and U waves (28%) [49]. Atrial fibrillation, supraventricular tachycardia, ventricular ectopic beats, ventricular tachycardia, and sinus tachycardia are the most common arrhythmias after AIS [41]. Conduction abnormalities are usually coupled with acute hemodynamic instability, which is associated with increased morbidity and mortality after AIS [5]. Furthermore, a history of heart failure, AIS severity, QTc interval, and ventricular extrasystoles are independent risk factors for serious cardiac events after AIS [35].

##### Ischemic myocardial injury and electrocardiographic abnormalities

The primary causative factors of stroke-induced myocardial dysfunction might be autonomic dysregulation and the physiological stress response [41]. Studies have shown that 18–70% of patients with major AIS will have coronary artery disease, regardless of previous cardiac history [50, 51]. These high rates might be explained by the fact that risk factors for cardiovascular and cerebrovascular disease are similar [52]. Additionally, ischemic myocardial damage occurs mainly within the first 24 h after AIS [53]. Concerning electrocardiographic changes, atrial fibrillation, atrial ventricular block, ST elevation, ST depression, and inverted T waves are not associated with outcomes, whereas tachycardia is significantly predictive of 3 months mortality [10]. Upright T waves and inverted T waves are four times more common, and ST segment depression is seven to 10 times more common in AIS patients than in age-matched peers without stroke [54]. In summary, early electrocardiographic monitoring and analysis of cardiac biomarkers are essential within the first 24 h—and possibly thereafter—to identify patients at risk for acute cardiac events, which are associated with poor clinical neurological outcome (Tables S2 and S3, Additional file 1). We therefore recommend continuous electrocardiographic monitoring, optimization of electrolyte balance, and serial troponin sampling in the AIS population.

#### Chronic cardiac dysfunction after AIS

Chronic cardiac dysfunctions after AIS are triggered by endothelial inflammation, oxidative stress, and catecholamine release leading to myocardial remodeling. Sympathetic stimulation activates a catecholamine surge, which causes vasoconstriction of the peripheral vessels and of the coronary arteries, followed by acute ischemia. The direct toxic effect of catecholamines on the myocardium and subsequent neurogenic hypertension also contribute to chronic myocardial dysfunction and remodeling [55].

##### Left ventricular systolic and diastolic dysfunctions

The current understanding of heart disease corroborates that cardiac remodeling is a common consequence of chronic myocardial or arrhythmic dysfunctions [56]. A pre-clinical study demonstrated that focal AIS and subsequent catecholamine release are followed by long-term cardiac dysfunction and remodeling [56, 57]. In this setting, metoprolol was tested and found to slow down cardiac remodeling and inhibit sympathetic activity [57]. Clinical studies are needed to confirm its utility and applicability as a protective agent in AIS. A recent study revealed that patients with atrial fibrillation of undetermined cause develop atrial fibrosis more frequently than those whose atrial fibrillation has a recognized cause, supporting the theory that chronic atrial remodeling could be associated with AIS [58]. Takotsubo cardiomyopathy is an example of left ventricular dysfunction that involves the apex and is associated with changes in cardiac biomarkers and electrocardiographic tracing. Because it is induced by neurogenic activation, it is largely reversible and normally self-limiting; clinical management is based on supportive care alone [44]. Takotsubo-like myocardial dysfunction affects mostly women and is associated with short-term poor functional outcomes, high mortality, and neurological deterioration [44]. Diastolic and systolic ventricular dysfunctions are also among the major complications of AIS [59, 60]. Cardiac dysfunction and its association with the outcome are described in Table S2, Additional file 1. In summary, early electrocardiographic and echocardiographic monitoring may help identify AIS patients at a high risk of chronic cardiac dysfunction.

##### Cerebral autoregulation: between blood pressure and hemodynamics

Cerebral autoregulation plays an essential role in maintaining cerebral blood flow and cerebral perfusion pressure within constant ranges to ensure adequate irrigation of the brain [61]. However, AIS can dysregulate this vulnerable system, leading to secondary brain damage [61]. Blood pressure control plays a pivotal role in the prevention of both AIS and post-AIS secondary brain damage [46, 61]. The main mechanisms involved in hypertension after AIS are still unclear, although the same mechanisms responsible for other cardiac complications have been proposed and may account for the increase in systolic blood pressure (> 140 mmHg) [61]. Among AIS subtypes, the lacunar infarct is the most closely associated with hypertension, suggesting an important role of the small lacunar vessels [62]. The main problem in these patients is not autoregulation itself but achieving a balance between the need for perfusion and the risk of hemorrhage. Thus, clinicians should seek good hemodynamic targets, albeit in the context of a failing heart. Blood pressure control is essential in AIS patients, especially in those with preexisting severe hypertension. Several studies have investigated the role of blood pressure control in AIS (Table S3, Additional file 1). According to their findings, both lower (120–150 mmHg) and higher (150–200 mmHg) systolic blood pressures are associated with increased risk of death [63]; indeed, the latest guidelines suggest keeping blood pressure below 180/105 mmHg in AIS patients who are eligible for emergency reperfusion therapy and in the post-thrombolytic phase [64]. Antihypertensive agents have been found to reduce the occurrence of recurrent AIS and cardiovascular events, but they did not affect the incidence of myocardial infarction or mortality [65]. Moreover, greater systolic blood pressure variability was associated with an increased risk of stroke recurrence [38], neurologic deterioration [66], and poor long-term functional outcome [67]. A meta-analysis of 12,703 patients showed that blood pressure lowering in the early phase of AIS did not prevent death [68] and is not recommended [69]. Several rationales have been offered for blood pressure management, suggesting that antihypertensive intervention is warranted in AIS if systolic blood pressure is > 180–230 mmHg or diastolic blood pressure is > 105–120 mmHg, thus avoiding important blood pressure variability [64]. In short, blood pressure control should be individualized, based on the patient’s clinical status and physiological response. There is no evidence regarding the best drug to use [64].

## Cardiovascular monitoring and treatment options

### Cardiovascular monitoring in patients at risk for AIS

Given the fact that cardiovascular and cerebrovascular diseases share similar risk factors, the Framingham Risk Score and the Pooled Cohort Equations [15, 32] should be considered when monitoring patients at risk for AIS and cardiovascular events. Moreover, a ratio between arterial systolic blood pressure at the ankle and brachial artery (known as the ankle–brachial index) higher than 0.9 indicates peripheral artery disease [32]. In patients with atrial fibrillation, the CHADS_2_ and CHA_2_DS_2_-VASc scores have been validated to identify the risk of AIS [45].

### Cardiovascular monitoring in patients with overt AIS

**Fig. 6:**
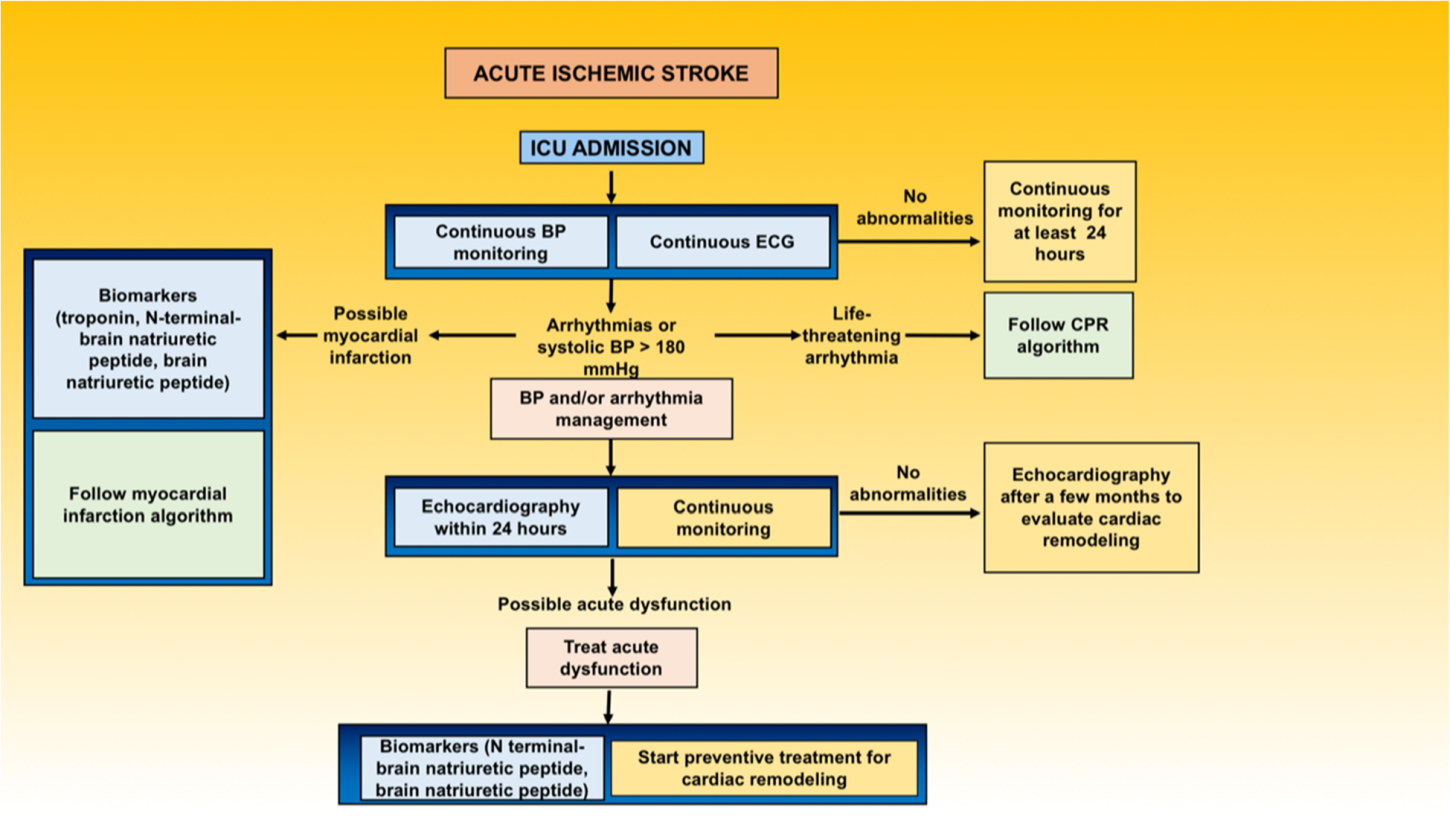
Flow chart of stroke management after intensive care unit admission. Summary of the first steps for the detection and management of possible cardiac complications in stroke patients after intensive care unit admission. Due to the lack of conclusive data, no specific recommendations on pharmacotherapy are given; readers are strongly advised to follow local protocols and international guidelines. ECG, electrocardiogram; BP, blood pressure; ICU, intensive care unit; CPR, cardiopulmonary resuscitation

General management of AIS is often complicated because of both possible cardiovascular dysfunction and several medical complications, such as swallowing impairment, neurogenic respiratory failure, stroke-associated pneumonia, gut dysbiosis, systemic inflammation, and need for tracheostomy or mechanical ventilation [41]. The National Institutes of Health Stroke Scale/Score (NIHSS) should be used for risk stratification, given its proven utility in detecting AIS patients at high cardiovascular risk; studies have found that patients with a NIHSS > 10 have higher troponin levels and more ischemic changes when compared to those with a score < 10 [70]. Figure 6 provides a practical flowchart for the management of AIS patients at risk of cardiovascular dysfunction in the intensive care unit (ICU).

#### Vital signs monitoring

Criteria for ICU admission vary significantly across countries and centers. In more severe cases, ICU admission of AIS patients might be essential to ensure invasive neuro- and cardiovascular monitoring and for early detection of any multiorgan complications [71]. Continuous cardiac monitoring is essential to detect severe cardiac arrhythmias and prevent sudden cardiac death. Moreover, within the first 24 h after an AIS, it might prevent cardiovascular complications (Fig. 6).

### Biomarkers

Cardiac enzymes may increase after AIS, but generally less than during a primary heart attack [72]. Troponin T is associated with AIS severity, insular involvement, and worse short-term prognosis [73, 74] (Table S4, Additional file 1). Plasma brain natriuretic peptide is also linked to worse outcomes in AIS patients [75–77]. Measurement of cardiac enzymes might be paramount for earlier identification of the patients at high risk of cardiovascular events after AIS. C-reactive protein (CRP) is involved in inflammatory and immune responses. A high-sensitivity CRP level greater than 2 or 3 mg/L could be a useful threshold to detect patients at increased cardiovascular risk [32].

#### Other considerations

Catheter ablation has been considered as a more effective alternative than anti-arrhythmic drugs to reverse atrial fibrillation to sinus rhythm [78]. The left atrial appendage is regarded as a major source of embolic manifestations after atrial fibrillation. Indeed, it has been identified as an important target for ablation [79] in patients who have contraindications to long-term oral anticoagulants, although this recommendation still needs to be clarified [80]. Nevertheless, electric isolation of the left atrial appendage has been associated with AIS occurrence [81]. Anticonvulsant agents, neuroleptics, and many other drugs commonly used in the ICU may prolong the QTc interval [82]; particular attention to this possible adverse effect must be paid in AIS patients. It is generally agreed that beta blockers are the cornerstone of medical therapy to reduce sympathetic hyperactivity and prevent cardiac remodeling after myocardial infarction [83], but the literature is inconclusive concerning their use in AIS patients. Beta blockers and alpha-2 antagonists demonstrated positive results for acute treatment of hypertension, while angiotensin-converting enzyme inhibitors and angiotensin II receptor blockers are good options for chronic blood pressure control after AIS [82].

## Conclusion

Despite great strides in our understanding of the brain–heart crosstalk after AIS, the roles of sympathetic control, gut dysbiosis, and inflammatory response are still under-investigated. Stroke-heart interactions are now a central theme of interest for researchers, and the causative mechanisms of stroke-related heart dysfunction are attractive new targets for future therapeutic strategies. Further studies are warranted to explore these fascinating theories and mechanisms and to translate pathophysiological knowledge into clinical practice.

## Supplementary information

**Additional file 1: Table S1.** Incidence and prevalence of all cardiac dysfunctions after ischemic stroke; **Table S2.** Cardiac dysfunctions after ischemic stroke and their association with outcome; **Table S3.** Blood pressure control in ischemic stroke and its association with outcome; **Table S4.** Troponin T as acute biomarker of cardiovascular complications after ischemic stroke.

## Data Availability

Not applicable.

## Abbreviations

AIS: Acute ischemic stroke
Akt: Protein kinase
BBB: Blood–brain barrier
FOXO: Forkhead box O
HPA: Hypothalamic–pituitary–adrenal axis
ICU: Intensive care unit
NIHSS: National Institutes of Health Stroke Scale
CRP: C-reactive protein
TMAO: Trimethylamine N-oxide
