# Supplementary material for: Brain–heart interaction after acute ischemic stroke
Source: Crit Care. 2020 Apr 21;24:163. doi: 10.1186/s13054-020-02885-8 (PMC7175494; doi:10.1186/s13054-020-02885-8)
Supplement: Supplementary file 1 — Additional file 1: Table S1. Incidence and prevalence of all cardiac dysfunctions after ischemic stroke; Table S2. Cardiac dysfunctions after ischemic stroke and their association with outcome; Table S3. Blood pressure control in ischemic stroke and its association with outcome; Table S4. Troponin T as acute biomarker of cardiovascular complications after ischemic stroke. [file 13054_2020_2885_MOESM1_ESM.docx]

**Additional Files**

**Brain-heart interaction after ischemic stroke**

Denise Battaglini^1,2^, Chiara Robba^1^, Adriana Lopes da Silva^3^, Cynthia dos Santos Samary^3^, Pedro Leme Silva^3^, Felipe Dal Pizzol^4^, Paolo Pelosi^1,2^, Patricia Rieken Macedo Rocco^3,5^

^1^Anesthesia and Intensive Care, San Martino Policlinico Hospital, IRCCS for Oncology and Neurosciences, Genoa, Italy

# ^2^Department of Surgical Sciences and Integrated Diagnostics, University of Genoa, Genoa, Italy

^3^Laboratory of Pulmonary Investigation, Carlos Chagas Filho Institute of Biophysics, Federal University of Rio de Janeiro, Rio de Janeiro, Brazil

^4^Unidade Acadêmica de Ciências da Saude, Universidade do Extremo Sul Catarinense (UNESC), Santa Catarina, Brazil

^5^Rio de Janeiro Network on Neuroinflammation, Carlos Chagas Filho Foundation for Supporting Research in the State of Rio de Janeiro (FAPERJ)

**Table S1.** **Incidence and prevalence of all cardiac dysfunctions after ischemic stroke.**

Studies are presented from most to least recent; only observational and randomized studies were included.

| **Autonomic dysfunction** | | | |
| --- | --- | --- | --- |
| **Author** | **Year** | **Journal** | **Incidence and prevalence** |
| Xiong et al.[1] | 2018 | Stroke | Based on the Ewing battery, minor autonomic dysfunction was identified in 24.0% of IS patients and significant autonomic dysfunction in 76.0% of IS patients. |
| Nayani et al.[2] | 2016 | Clin Neurol Neurosurg | Overall, 21.8% of patients developed autonomic dysfunction. |
| Idiaquez et al.[3] | 2015 | Clin Neurol Neurosurg | Orthostatic hypotension occurred in 3.6% of IS patients and 4.4% of controls. All patients had hypertension. |
| Xiong et al.[4] | 2012 | Clin Neurol Neurosurg | The prevalence of severe autonomic dysfunction in ischemic stroke patients was 76.5%. |
| Diserens K et al.[5] | 2006 | Eur J Neurol | Acute autonomic dysfunction was present in 71% of patients. |
| **Arrhythmias** | | | |
| **Author** | **Year** | **Journal** | **Incidence and prevalence** |
| Tanislav et al.[6] | 2019 | Eur J Neurol | Atrial fibrillation at 5-year follow-up occurred in 10.4% of stroke patients vs 4.8% of controls (HR 4.95; 95%CI 1.93-2.09, p<0.001). |
| Hsieh et al.[7] | 2018 | J Neurol Neurosurg Psichiatry | Atrial fibrillation was detected in 481 patients (41.4%) before IS and in 680 patients (58.6%) after IS. |
| Wachter et al.[8] | 2017 | Lancet Neurol | Atrial fibrillation was found after 6 months in 14% of 200 patients of the enhanced and prolonged monitoring group vs 5% in the standard care group. |
| Adeoye et al.[9] | 2017 | Glob Heart | ECG abnormalities were noted in 85.4% of IS patients. HS was associated with less atrial fibrillation than IS (1 vs 6.7%, p=0.002), but more left ventricular hypertrophy (64.4 vs 51.4%, p=0.004). |
| Hromadka et al.[10] | 2016 | J Stroke Cerebrovasc Dis | Incidence of prolonged QTc was 65.2% at baseline and 26.1% after 48 hours. |
| Fernandez-Menendez et al.[11] | 2016 | Neurologia | Cardiac arrhythmias occurred in 29.5% of IS patients. Tachyarrhythmia (ventricular tachyarrhythmias, supraventricular tachyarrhythmias, complex ventricular ectopy) occurred in 27.1% of patients, and bradyarrhythmia occurred in 3.91%. |
| Yayehd et al.[12] | 2015 | Arch Cardiovasc Dis | Overall, 1.85% of patients showed paroxysmal AF lasting >30 seconds during monitoring, 1.85% had paroxysmal AF lasting < 30 seconds, 3.7% showed numerous premature atrial complexes, and 1.85% showed non-sustained ventricular tachycardia. |
| Bobinger et al.[13] | 2015 | Clin Res Cardiol | Early repolarization pattern was found in 8.04% of stroke patients. Early repolarization was more frequent among patients affected by intracerebral and subarachnoid hemorrhage (13.0%) than those affected by IS (7.0%; p=0.024). QRS duration (OR 0.97, 95 %CI 0.95–0.99, p=0.012), QT duration (OR 1.01, 95 %CI 1.00–1.01, p=0.001), and mechanical ventilation on admission (OR 0.32, 95 %CI 0.14–0.75, p=0.009) were independent predictors of early repolarization. |
| Purushothaman et al.[14] | 2014 | J Nat Sci Biol Med | Electrocardiographic changes were noted in 78% of patients (both IS and HS). T-wave inversion was noted in 34.48%, ST-segment depression in 32.75%, QTc prolongation in 29.31%, and U waves in 27.58%. |
| Gonzalez-Toledo et al.[15] | 2013 | J Stroke Cerebrovasc Dis | New atrial fibrillation was seen in 8.36%, known atrial fibrillation in 23.27%, and normal sinus rhythm in 68.36%. |
| Kallmunzer et al.[16] | 2012 | Stroke | Significant cardiac arrhythmias occurred in 25.1% of IS patients. Tachycardia was more frequent than bradycardia. |
| Ritter et al.[17] | 2011 | BMC Neurol | Tachycardia was found in 5% of IS patients, and bradycardia in 5%. |
| Christensen et al.[18] | 2005 | J Neurol Sci | Electrocardiographic abnormalities: 60% in IS, 50% in ICH, 44% in TIA. |
| Dogan et al.[19] | 2004 | Anadolu Kardiyol Derg | Ischemic electrocardiographic changes were found in 65% of IS and 57% of HS patients (p=0.33). Atrial fibrillation was more frequent in IS (34%) than HS (13%) (p=0.01). |
| Li et al.[20] | 1999 | Zhonghua Nei Ke Za Zhi | Supraventricular arrhythmias were more frequent in patients with insular lesions than in controls. Right insular lesions were more associated with supraventricular arrhythmias, while left insular infarction was associated with ST segment abnormalities. |
| **Myocardial infarction and coronary artery disease** | | | |
| **Author** | **Year** | **Journal** | **Incidence and prevalence** |
| Bhatia et al.[21] | 2019 | J Stroke Cerebrovasc Dis | Of 300 IS patients, 247 were asymptomatic, of whom 4.81% had a positive myocardial perfusion scan. The overall prevalence of CAD was 17.67%. |
| Alqahtani et al.[22] | 2017 | Stroke | Among 864,043 IS cases, 1.6% had acute myocardial infarction (79.5% NSTEMI and 20.5% STEMI). |
| Mathias et al.[23] | 2014 | J Cardiovasc Dis | Of 426 IS patients, 4.9% had myocardial infarction. |
| Gattringer et al.[24] | 2014 | Cerebrovasc Dis | Only 1% of 406,603 IS patients and 0.3% of ICH patients had myocardial infarction. Anterior circulation and left-sided stroke were more frequent in patients with myocardial infarction. |
| Cha et al.[25] | 2013 | Eur J Neurol | Overall, 70.4% of 1,733 IS patients had CAD (clinically significant in 33.3%). The presence of CAD increased with CHADS2 and CHA2 DS2 scores (p<0.001). |
| Kim et al.[26] | 2012 | Eur J Neurol | Among 443 IS patients who underwent multidetector cardiac computed tomography, 36% showed asymptomatic CAD; invasive coronary angiography was performed in 10.5%. Ultimately, 5.0% patients underwent coronary intervention. |
| Micheli et al.[27] | 2012 | J Neurol | Among 814 patients (84.2% with IS), 2.25% presented with myocardial infarction, 4.85% developed acute heart failure, and 2.08% had both myocardial infarction and heart failure. |
| Jensen et al.[28] | 2012 | Int J Cardiol | Among 392 patients (24 IS, 368 acute chest pain), those with IS had higher odds of coronary artery plaque (OR 4.9, p<0.01) than those without IS. If stratified by extent of plaque, the IS group had a more than 18-fold increase in risk of having >4 affected segments than patients without IS (OR 18.3, p<0.01). |
| Amarenco et al.[29] | 2011 | Stroke | Coronary plaques were found in 61.9% of 864,043 IS patients at angiography, and coronary stenosis in 25.7%. The more territories were involved, the more plaques were found. |
| Cho et al.[30] | 2011 | Cerebrovasc Dis | Of 469 patients with IS (274 with no history of CAD), 22.3% had asymptomatic CAD with more than 50% stenosis; 1.2% had complicated aortic plaques. |
| Yoon et al.[31] | 2011 | Int J Cardiovasc Imaging | Of 175 patients with IS, 60% had demonstrable atherosclerotic plaques; 21% had occult CAD with >50% stenosis. Only coronary calcium score predicted occult CAD by coronary computed angiography. |
| Calvet et al.[32] | 2010 | Circulation | The prevalence of asymptomatic CAD >50% was 18% (95%CI, 14-23). |
| Arauz et al.[33] | 2010 | Clin Neurol Neurosurg | Among 125 patients with IS, silent CAD was identified in 32%, of whom 30% had single lacunar infarction, 33% had multiple lacunar infarction, and 33% had a large-vessel stroke. The stroke recurrence rate was 8%. |
| Liao et al.[34] | 2009 | Eur J Neurol | Among 9,180 IS patients, 2.3% had myocardial infarction. |
| Hoshino et al.[35] | 2008 | Intern Med | Of 100 patients with IS, 36 had CAD. |
| Lee et al.[36] | 2008 | Neurocrit Care | Of 1,357 IS patients, 0.9% developed myocardial infarction. Five patients died of cardiac complications. |
| Seo et al.[37] | 2008 | Eur Neurol | Coronary artery stenosis was detected in 25.4% of 89 patients with IS. Intracranial arterial stenosis was not associated with coronary stenosis. |
| Nighoghosian et al.[38] | 2006 | Eur Neurol | Dobutamine stress echocardiography was positive in 15% of 64 patients with IS. The main predictive factor for a positive dobutamine stress test was aortic arch atheroma (p=0.003). |
| Leys et al.[39] | 2006 | Cerebrovasc Dis | Among 753 patients with IS, 16% had had a previous coronary event, 31% a previous stroke or TIA, and 15% had peripheral artery disease. Atherothrombosis was found in 47.5% of patients (33.7% coronary artery, 16.6% aortic atheroma, and 22.7% peripheral artery disease). |
| Chimowitz et al.[40] | 1997 | Stroke | Among 69 patients with IS or TIA, 50% of those with large-artery vascular disease had an abnormal stress test, versus 23% of those with other brain ischemia (p=0.04). 60% of patients with large-artery vascular disease and an abnormal stress test had CAD, versus 25% of those with other causes of brain ischemia. Patients with an abnormal stress test were more likely to smoke (p=0.006), have large-artery cerebrovascular disease (p=0.02), be older (p=0.02), and have left ventricular hypertrophy (p=0.07). |
| Di Pasquale et al.[41] | 1988 | Eur Heart J | Among 303 patients with a positive maximal exercise treadmill test undergoing myocardial scintigraphy (190 IS, 113 control), 26% of those with IS had a positive test, versus only 6% of controls (p<0.01). |
| **Cardiac dysfunction and remodeling** | | | |
| Choi et al.[42] | 2017 | Neurology | Left ventricular wall motion abnormalities occurred in 9.96% of patients with IS. |
| Kim et al.[43] | 2016 | J Stroke Cerebrovasc Dis | Among 1554 patients with acute IS, normal left ventricular function was identified in 91.18%, mild left ventricular dysfunction in 5.6%, and severe left ventricular dysfunction in 3.22%. |
| Burkot et al.[44] | 2015 | J Card Fail | Among 566 patients with stroke, decompensated heart failure was diagnosed in 17%. Of these, 57% had preserved ejection fraction. |

*IS, ischemic stroke; HS, hemorrhagic stroke; AF, atrial fibrillation; OR, odds ratio; CI, confidence interval; ICH, intracranial hemorrhage; TIA, transient ischemic attack; CAD, coronary artery disease; STEMI, myocardial infarction with ST segment elevation; NSTEMI, myocardial infarction without ST segment elevation*

**Table S2. Cardiac dysfunctions after ischemic stroke and their association with outcome**

Studies are presented from most to least recent; only observational and randomized studies were included.

| **Autonomic dysfunction** | | | | | | |
| --- | --- | --- | --- | --- | --- | --- |
| **Author** | **Year** | **Journal** | **Type of study** | **Number of patients** | **Primary endpoint** | **Results** |
| Verma et al.[45] | 2019 | Brain Sci | Observational, prospective | 70 (41 IS, 29 control) | To compare heart rate variability and pulse rate variability in IS patients. | The stroke group had a lower value for electrocardiographic RR intervals (heart rate variability) in the seated (p<0.001) and standing (p<0.001) position with respect to controls. Pulse arrival time (arterial stiffness) was 248±7 ms vs. 270±8 ms (p<0.05), suggesting higher arterial stiffness in stroke survivors with respect to controls in the standing position. |
| Tobaldini et al.[46] | 2019 | J Clin Med | Observational, prospective | 41 IS | To assess the prognostic value of heart rate variability in acute IS patients to understand parasympathetic modulation. | A prevalent parasympathetic modulation (low frequency) was found in patients with NIHSS ≥14. Right-sided strokes were associated with higher respiratory vagal control (high frequency). |
| Grilletti et al.[47] | 2018 | Clinics | Observational, prospective | 17 (10 IS, 7 control) | To assess cardiovascular autonomic modulation in chronic IS patients. | No differences were found in systolic and diastolic pressure or heart rate between groups. The IS group had lower indexes for heart rate variability and a lower high-frequency band for heart rate variability with respect to the control group. Systolic blood pressure variability was higher in the IS group. |
| He et al.[48] | 2018 | J Neurol | Observational, prospective | 516 IS | Association of HR variability by fractal dimension with neurological deterioration (within 72 hours) and recurrent IS (1 year later). | Associations were found between HR variability by fractal dimension ≤ 1.05 and neurological deterioration (OR 2.64; 95%CI 1.55–4.49; p<0.001), and between HR variability by fractal dimension ≤ 1.15 and recurrent IS (OR 5.40; 95%CI 3.02–9.64; p<0.001). Therefore, fractal dimension ≤ 1.05 and ≤ 1.15 were independently associated with neurological deterioration and recurrent IS. |
| Xiong et al. [1] | 2018 | Stroke | Observational, prospective | 150 IS | To evaluate whether post-stroke autonomic dysfunction (assessed by Ewing’s battery test) can predict clinical outcome (by mRS). | Poor functional outcome was detected in 32.5% of the significant autonomic dysfunction group vs 13.9% of the minor autonomic dysfunction group (p=0.031). Magnitude of autonomic dysfunction was an independent predictor of unfavorable outcome (OR 3.26, 95%CI 1.141–9.335; p=0.027). |
| Nozoe et al.[49] | 2018 | Eur Neurol | Observational, prospective | 21 IS | Comparison in heart rate variability between patients with neurological vs non-neurological disorders. | Authors divided patients into two groups: neurological disorders (7 patients) and non-neurological disorders (14 patients). No differences were found between the two groups in blood pressure (systolic, diastolic), heart rate, or high frequency, at rest or during mobilization. Differences were found in low frequency/high frequency ratio only during mobilization (p=0.03). |
| Ha et al.[50] | 2018 | Acta Neurol Scand | Observational, prospective | 60 IS | The primary outcome was neurological progression, and the secondary endpoint was outcome at 3 months (NIHSS). IS patients underwent autonomic function tests (30° head-up tilt test, Valsalva test, heart rate response after deep breathing, and sympathetic skin response). | Abnormal blood pressure response in the Valsalva test was associated with IS (acute lacunar infarction) and neurological progression, leading to unfavorable 3-month outcome. |
| Rodriguez et al.[51] | 2017 | Medicine | Observational, prospective | 23 (10 IS, 13 control) | To compare autonomic variables (arterial blood pressure by plethysmography, muscle-pump baroreflex by electromyography, heart rate variability by 3-lead ECG, and cerebral blood flow velocity by transcranial doppler) between IS patients and controls during sitting and standing for 5 minutes. | The IS group showed greater decline in the low frequency component of heart rate variability (p= 0.043). Overall, cardiovascular parameters and autonomic function did not differ between the two groups. |
| Nayani et al.[2] | 2016 | Clin Neurol Neurosurg | Observational, prospective | 101 IS | To evaluate whether autonomic dysfunction (assessed by Ewing’s battery test and heart rate variability with 24-hour Holter) is related to neurological and cardiovascular outcomes. | Stroke severity was associated with autonomic dysfunction (OR 2.12, 95%CI 1.43–2.71). Infarct volume was associated with autonomic dysfunction, whether detected clinically (p=0.05) or by Holter (p=0.001). Those with autonomic dysfunction had poorer outcomes and higher cerebrovascular recurrence at 3 months (p=0.01) and 1 year (p=0.01). |
| Xu et al.[52] | 2016 | Clin Interv Aging | Observational, prospective | 113 (63 IS, 50 control) | To assess the correlation between heart rate variability and neurological outcome (by NIHSS). | RR interval, deceleration capacity, acceleration capacity, and SD of normal-to-normal interval in the IS group were lower than in controls (p=0.003, p=0.002, p=0.006, and p=0.043, respectively). Correlation analysis showed that deceleration capacity, acceleration capacity, and SD of normal-to-normal interval correlated negatively with NIHSS score (*r*=−0.279, *r*=−0.266, and *r*=−0.319; p=0.027, p=0.035, and p=0.011). |
| Constantinescu et al.[53] | 2016 | Acta Neurol Belg | Observational, prospective | 80 (40 IS, 40 control) | To assess cardiac autonomic activity in patients with IS in the middle cerebral artery territory. | Heart rate variability parameters were modified in IS patients versus those without IS (p<0.05). Parasympathetic predominance was greater in left versus right hemisphere stroke (p<0.01). |
| Idiaquez et al.[3] | 2015 | Clin Neurol Neurosurg | Observational, prospective | 100 (5 IS, 55 control) | To assess autonomic dysregulation in patients with hypertension (IS and control). | Scale for Outcomes in Parkinson disease-Autonomic (SCOPA-AUT) scores differed between IS and controls (p=0.001). |
| Erdur et al.[54] | 2014 | Int J Cardiol | Observational, prospective | 1,335 IS | To assess the effect of heart rate at admission on outcome. | Heart rate ≥ 83 bpm was independently associated with in-hospital mortality when compared to HR ≤ 69 bpm (aOR 4.42, 95%CI 1.36–14.42, p=0.01). Relative risk for in-hospital death was elevated by 40% for every additional 10 bpm (p=0.003). |
| Graff et al.[55] | 2013 | J Hypertens | Observational, prospective | 75 IS | To assess the effect of heart rate variability (by ECG changes) and entropy values on outcome (mRS, NIHSS). | 16% of patients had poor early (7-day) outcome, while 18% had poor late (90-day) outcome. In poorer-outcome patients, sample entropy and fuzzy entropy were lower, reflecting lower complexity of the heart rhythm (p=0.053 for fuzzy entropy). |
| Xiong et al.[56] | 2013 | Int J Stroke | Observational, prospective | 131 (94 IS:34 acute, 60 chronic, and 37 control) | To investigate whether autonomic function impairs during different phases in IS patients (by Ewing’s battery test). | Heart rate variability in IS patients was associated with impairment in Valsalva ratio (p=0.002) and heart rate response to deep breathing (p<0.001) in the acute phase, as well as impairment in all parasympathetic tests (all p<0.05) in the chronic phase, in comparison with controls. |
| Xiong et al.[4] | 2012 | Clin Neurol Neurosurg | Observational, prospective | 34 IS | To evaluate whether autonomic dysfunction (assessed by Ewing’s battery test) is related to functional outcome (assessed by Barthel index) 2 months after IS. | 2 months after stroke onset, Barthel index increased in patients with minor autonomic dysfunction and severe autonomic dysfunction. The mean Barthel index score onset and its delta at 2 months were lower in patients with severe autonomic dysfunction than in those with minor autonomic dysfunction (p<0.05). |
| Gasecki et al.[57] | 2012 | Atherosclerosis | Observational, prospective | 134 IS | Association between carotid-femoral pulse wave velocity and central augmentation index on NIHSS. | Low aortic stiffness (p<0.0001), but not central augmentation index, was significantly associated with early favorable outcome after adjustment for age, NIHSS, blood glucose level, heart rate, systolic and mean blood pressure on admission (OR 0.17, 95%CI (0.05-0.60); p=0.006 (at 7 days). |
| Chen et al.[58] | 2011 | Kaohsiung J Med Sci | Observational, prospective | 126 IS | To assess cardiac autonomic function in IS patients by measuring heart rate variability. | The low- and high-frequency components of heart rate variability in IS patients were lower than in controls. No differences were found between large artery atherosclerosis and small-vessel occlusion, but patients with small-vessel occlusion showed increased sympathetic and reduced vagal activities. |
| Bassi et al.[59] | 2007 | Eur J Neurol | Observational, prospective | 85 IS | To assess the impact of cardiac autonomic dysfunction on functional outcome after a rehabilitation program in patients with recent ischemic stroke. | Patients underwent 24-h Holter monitoring before the start of a 60-day rehabilitation program. Unfavorable outcome and disability were found in 44.7% of patients (Barthel). Normal-to-normal R wave to R wave RR interval (OR 9.67, 95%CI 2.58–18.67, p=0.006) was independently associated with unfavorable functional outcome. |
| Diserens et al.[5] | 2006 | Eur J Neurol | Observational, prospective | 100 IS | To evaluate acute autonomic dysfunction on the hemi-body contralateral to the lesion in the acute phase of stroke. | Acute autonomic dysfunction was positively associated with lesions in the post-central cortex (p=0.037), internal capsule (p=0.005), basal ganglia (p=0.002), or insula (p=0.011), and negatively associated with lesions in the brainstem (p=0.004). Autonomic dysfunction was associated with sensory deficits (p=0.001) and contralateral hyperkinesia (p=0.004). |
| Meyer et al.[60] | 2004 | Neuroreport | Observational, prospective | 29 IS (15 left IS, 14 right IS) | To assess autonomic dysfunction in patients with ischemic insular versus non-insular cortex infarction, and to evaluate a possible lateralization of autonomic activity. | Norepinephrine and epinephrine values were used to assess autonomic function. Sympathetic activity was higher in insular than in non-insular infarction (p<0.05). Hyperactivation of the sympathetic nervous system was predominant in right stroke involving the insular cortex (p<0.05). |
| Strittmatter et al.[61] | 2003 | Eur Neurol | Observational, prospective | 39 IS (19 left IS, 14 right IS, 6 brainstems/  cerebellar) | To assess autonomic dysfunction in patients with ischemic stroke, and to evaluate a possible lateralization of autonomic activity. | The authors used norepinephrine and epinephrine values to assess autonomic function. IS was associated with increased sympathetic activity in all groups, with decreased norepinephrine in left IS (p<0.01) and brainstem/cerebellar IS (p<0.05). Norepinephrine was higher in right IS than in brainstem/cerebellar IS (p<0.05). Autonomic function was altered by an elevation in cardiovascular parameters, mainly in right stroke. |
| Sander et al.[62] | 2001 | Neurology | Observational, prospective | 112 IS | To assess the prognostic impact (by Barthel index and mRS) of early sympathetic activation after IS (by norepinephrine levels greater than 300 pg/mL, nighttime blood pressure increases, and insular involvement). | Norepinephrine, nighttime blood pressure, and insular involvement were associated with a lower Barthel index (p<0.005) at 1-year follow-up. Insular infarction, norepinephrine concentration, right-sided infarction, and nighttime blood pressure were independent predictors of unfavorable functional outcome. Moreover, there was a higher rate of cardiovascular and cerebrovascular events (HR 2.9; 95%CI, 1.07-6.83; p<0.04) in those with increased norepinephrine levels. |
| Korpelainen et al.[63] | 1996 | Stroke | Observational, prospective | 62 (31 Is, 31 controls) | To assess heart rate variability in IS patients and controls. | Standard deviation of RR intervals (p<0.001), total power (p<0.0001), very-low-frequency power (p<0.0001), low-frequency power (p<0.001), and high-frequency power (p<0.05) were lower than in controls in both acute and sub-acute phases. Heart rate variability dysfunction correlated with severity of neurological outcome and disability. |
| **Arrhythmias** | | | | | | |
| Krawczyk et al.[64] | 2019 | EP Eur | Observational, prospective | 9,791 IS | To assess association between post-stroke AF and dementia (comparison among AF diagnosed during admission, after discharge, or no AF) | Dementia risk was higher for patients in whom AF occurred during admission (HR 1.78, 95%CI 1.51–2.10) and after discharge (HR 1.74, 95%CI 1.47–2.05) relative to no AF. |
| Tanislav et al.[6] | 2019 | Eur Neurol | Observational, prospective | 45,548 IS | To detect AF (stroke patients and controls). | AF at 5-year follow-up in stroke vs controls: 10.4% vs 4.8% (HR 4.95, 95%CI 1.93-2.09, p<0.001). |
| Hsieh et al.[65] | 2018 | Int J Cardiol | Observational, prospective | 1,161 IS | To assess whether AF diagnosed before or after IS correlates with outcome. | Patients with pre-IS AF had a higher prevalence of underlying heart diseases than those with post-IS AF (67.2% versus 39.0%, p<0.001). Patients with pre-IS AF showed a higher risk of the composite outcome than those with post-IS AF (HR 1.42, 95%CI: 1.13–1.79, p=0.003). |
| Wachter et al.[8] | 2017 | Lancet Neurol | RCT | 398 IS (200 enhanced and prolonged monitoring, 198 standard care) | To assess whether enhanced and prolonged monitoring reduce the occurrence of atrial fibrillation with respect to standard care. | After 6 months, atrial fibrillation was found in 14% of 200 patients in the enhanced and prolonged monitoring group vs 5% in the standard-care group (95%CI 3.4–14.5, p=0.002). |
| Dahlin et al.[66] | 2017 | Medicine | Observational, prospective | 215 (150 IS and 65 HS) | To examine the association between ventricular arrhythmia and in-hospital mortality. | Ventricular arrhythmia was associated with in-hospital mortality (OR 1.75, 95%CI 1.6-1.2). HS was associated with higher in-hospital mortality than IS (OR 9.0 95%CI 8.6-9.4). |
| Adeoye et al. [9] | 2017 | Glob Heart | Observational, prospective | 890 (583 IS, 307 HS) | To investigate the prevalence and prognoses of electrocardiographic abnormalities among acute IS in Africa. | Atrial enlargement was associated with severe disability and 1-month mortality (aOR 1.45, 95%CI 1.04-2.02). |
| Hromadka et al.[10] | 2016 | J Stroke Cerebrovasc Dis | Observational, prospective | 69 IS | To assess the association of prolonged QTc with neurological outcome and death. | Baseline QTc was not associated with neurological outcome (p=0.27). Prolonged QTc after 48 hours was associated with mRS at discharge (p<0.0001). Hospitalized patients who died had more frequently prolonged QTc after 48 hours (p<0.0001), higher troponin I (p=0.003), and higher BNP (p=0.014). |
| Fernandez-Menendez et al.[11] | 2016 | Neurologia | Observational, prospective | 332 IS | To assess incidence and prevalence of cardiac arrhythmias in IS patients. | Arrhythmias were independently associated with brain lesions of larger volume, as well as with older age. |
| Bobinger et al.[13] | 2015 | Clin Res Cardiol | Observational, prospective | 771 (IS and HS) | To assess the prevalence of early repolarization pattern in acute stroke patients, as well as functional outcome and mortality after 90 days. | Early repolarization pattern on admission was not associated with outcome or mortality after stroke (p=0.582). |
| Purushothaman et al.[14] | 2014 | J Nat Sci Biol Med | Observational, prospective | 100 (58 IS, 42 HS) | To assess whether incidence, prevalence, and mortality of IS and HS correlate with electrocardiographic changes. | A higher mortality rate was found in IS patients with ST-T changes (66.66%) and HS patients with positive U waves (60%). |
| Li et al.[67] | 2013 | J Stroke Cerebrovasc Dis | Observational, prospective | 1,297 IS or TIA | Outcome and death at 3-month follow-up in patients who developed AF after IS. | Each year of age (OR 1.031; 95%CI 1.017-1.045), NIHSS at admission (OR 1.219; 95%CI 1.185-1.254), and female gender (OR 1.710; 95%CI 1.296-2.256) were independent risk factors for poor outcome at 3 months after IS; independent risk factors for 3-month mortality included age (OR 1.024; 95%CI 1.007-1.041), NIHSS at admission (OR 1.122; 95%CI 1.100- 1.144), and history of heart failure (OR 1.855; 95%CI 1.141-3.015). |
| Gonzalez-Toledo et al.[15] | 2013 | J Stroke Cerebrovasc Dis | Observational, prospective | 275 IS | To assess whether new-onset AF can determine atrial enlargement, higher frequency of insular stroke, and higher brain infarct area. | New-onset AF patients had less left atrial enlargement (60.9% versus 91.2%, p=0.001), a smaller left atrial area (22.0 vs 26.0 cm^2^, p=0.021), and a higher frequency of insular IS (30.4% vs 9.5%, p=0.017) than those with known AF. Newly diagnosed AF was associated a higher volume of brain infarcts (60.9% vs 37.2%, p=0.029) and a higher rate of insular involvement (30.4% vs 7.3%, p=0.001) than sinus rhythm. |
| Kallmunzer et al.[16] | 2012 | Stroke | Observational, prospective | 501 IS | To assess incidence and prevalence of arrhythmias and their correlation with neurological outcome at admission. | Cardiac arrhythmias were independently associated with older age and neurological deficits (NIHSS on admission). |
| Ritter et al.[17] | 2011 | BMC Neurol | Observational, prospective | 256 IS | To assess incidence of tachycardia and bradycardia and observe cardiac rhythms; to assess the influence of risk factors on heart rate and on dependence (by mRS at 3 months) and mortality. | Neither tachycardia nor bradycardia independently predicted poor outcome at 3 months. Stroke location did not change rhythm. Clinical severity and age were the only predictors of poor outcome. |
| Stead et al.[68] | 2009 | J Stroke Cerebrovasc Dis | Observational, prospective | 480 IS | To assess the association between QTc and 90-day mortality and functional outcome after IS. | Patients with prolonged QTc were compared to patients with normal QTc. The estimated survival at 90 days was 70.5% and 87.1%, respectively (RR 2.5; 95%CI 1.5-4.1; p=0.001). Patients with a prolonged QTc showed poorer functional status than patients without a prolonged interval (OR 1.8; 95%CI 1.2-3.0; p=0.006). |
| Abboud et al.[69] | 2006 | Ann Neurol | Observational, prospective | 986 (493 IS, 493 control) | To assess whether IS or cardiac arrhythmias after IS are associated with 2-year all-cause or vascular death. | Acute stroke was independently associated with heart rate, abnormal repolarization, atrial fibrillation, and ventricular and supraventricular ectopic beats. Right insular IS was associated with 2-year all-cause death (HR 2.11; 95%CI 1.27-3.52) and vascular death (HR 2.00; 95%CI 1.00-3.93). |
| Colivicchi et al.[70] | 2005 | Stroke | Observational, prospective | 208 IS | To evaluate the prognostic implications of right-sided insular damage, cardiac autonomic derangement, and arrhythmias after acute IS. | The 1-year likelihood of death was OR 0.23; 95%CI, 0.17-0.30). Age (HR, 1.06; 95%CI, 1.01-1.10; p<0.0087), stroke severity on admission (HR, 1.25; 95%CI, 1.13-1.39; p<0.0001), presence of right-sided insular damage (HR, 2.01; 95%CI, 1.13-1.39; p<0.0187), RR interval (HR, 3.32; 95%CI, 1.67 to 6.24; p<0.002), and ventricular tachycardia (HR, 2.99; 95%CI, 1.58-5.67; p<0.0007) were independent predictors of 1-year mortality. |
| Colivicchi et al.[70] | 2004 | Stroke | Observational, prospective | 103 IS | To assess the effects of acute right insular ischemic damage on heart rate variability and arrhythmias. | Patients with right-sided insular stroke showed lower SD values of all RR intervals and of the root mean square of differences of adjacent RR intervals, and higher low-frequency/high-frequency ratio values (p<0.05). Right insular IS was associated with more complex arrhythmias than other localizations (p<0.05). |
| Christensen et al.[18] | 2005 | Stroke | Observational, prospective | 692 IS, 155 ICH, 223 TIA | To assess the effect of electrocardiographic abnormalities on outcome and 3-months mortality. | AF (OR 2.0, 95%CI 1.3– 3.1), atrioventricular block (OR 1.9, 95%CI 1.2–3.9), ST elevation (OR 2.8, 95%CI 1.3–6.3), ST depression (OR 2.5, 95%CI 1.5–4.3), and inverted T-waves (OR 2.7, 95%CI 1.6–4.6) were independent from outcome. In ICH, sinus tachycardia (OR 4.8, 95%CI 1.7–14.0), ST depression (OR 5.2, 95%CI 1.1–24.9), and inverted T-waves (OR 5.2, 95%CI 1.2–22.5) predicted poor outcome. Rapid heart rate predicted 3-month mortality (OR 1.7, 95%CI 1.02–2.7). |
| Afsar et al.[71] | 2003 | Arch Neurol | Observational, prospective | 36 IS | To assess if QT dispersion is predominant in acute stroke patients and could be related to lesion extent or localization. | QT dispersion, corrected QT dispersion, and automated QT dispersion were increased at 24 hours when compared to 72 hours (60 vs 40 milliseconds, p<0.005; mean [SD], 56 [19] vs 36 [21] milliseconds, p<0.001; and 50 vs 34 milliseconds, p<0.005). No differences in QT dispersion were found between stroke and control groups at 72 hours, whereas at 24 hours, corrected QT dispersion was greater in patients with large infarcts and large hemorrhages (mean [SD], 70 [20] vs 51 [20] milliseconds, P<0.05), and at 72 hours, in right vs left-sided lesions (mean [SD], 39 [18] vs 24 [18] milliseconds, p<0.05). |
| **Myocardial infarction and acute coronary syndrome** | | | | | | |
| Alkhachroum et al.[72] | 2019 | J Clin Neurosc | Observational, prospective | 1,655 (818 IS, 306 ICH, 169 SAH) | To assess the relationship between high troponin levels and outcome in stroke patients. | Troponin was elevated in 24.1% of IS, 27.1% of ICH, and 39% of SAH cases. In SAH, higher initial and peak troponin levels were associated with higher Hunt and Hess score (OR 4.2; 95%CI, 1.6-11.4, p=0.005 and OR 3.14; 95%CI, 1.5-6.5, p=0.002, respectively). Among patients with higher troponin, mortality was 14.7% in IS, 31.3% in ICH, and 43.8% in SAH. Higher troponin in IS patients were associated with higher mortality (OR 6.16; 95%CI, 2.46-15.4, p<0.001) and worse discharge disposition (OR 2.3; 95%CI, 1.19-4.45, p=0.01). |
| Alqahtani et al.[22] | 2017 | Stroke | Observational, prospective | 864,043 IS | To assess incidence and outcome of myocardial infarction in stroke patients. | In-hospital mortality was 21.4% in patients with myocardial infarction and 7.1% in patients without myocardial infarction (p<0.001). In-hospital length of stay and cost of care were 50% higher in the myocardial infarction group. |
| Kang et al.[73] | 2016 | J Stroke Cerebrovasc Dis | Observational, prospective | 12,227 IS | To estimate rates of recurrent stroke, myocardial infarction, and major vascular events during the first year after acute IS. | Recurrence of stroke at 30 days, 90 days, and 1 year was 2.7%, 3.9%, and 5.7% respectively. The rate of vascular events was 0.1%, 0.3%, and 0.5% for myocardial infarction and 8.1%, 10.6%, and 13.7% for major vascular events at the same time points. |
| Mathias et al.[23] | 2014 | J Cardiovasc Dis | Observational, prospective | 426 IS | To assess impact of myocardial infarction and pneumonia in IS. | IS patients with myocardial infarction had more severe strokes (p=0.014) and more frequent pneumonia (26% vs. 9%, p=0.004). In-hospital mortality for these patients was 3 times greater than in those without infarction (OR 3.2 95%CI 1.1-9.7, p=0.036). |
| Gattringer et al.[24] | 2014 | Cerebrovasc Dis | Observational, prospective | 46,603 IS | To assess the frequency, clinical characteristics, and outcome of stroke patients with acute myocardial infarction. | IS patients showed higher mortality (14.5 vs. 2%; p<0.001) and more complications, such as progressive stroke and pneumonia. Previous myocardial infarction and stroke severity at admission (by NIHSS) were independently associated with myocardial infarction occurrence. |
| Micheli et al.[27] | 2012 | J Neurol | Observational, prospective | 814 IS | To assess occurrence of myocardial infarction and acute heart failure during hospital stay and mortality after 3 months. | Of the included patients (84.2% with IS), 2.25% had myocardial infarction, 4.85% had acute heart failure, and 2.08% had both myocardial infarction and heart failure. At 3 months, 18.8% of patients had died. Among patients with cardiac events, 60.4% died, versus only 15.9% of those without cardiac events (p<0001). Acute myocardial infarction and/or acute heart failure were associated with increased mortality at 3 months (p=0.001). |
| Calvet et al.[32] | 2010 | Circulation | Observational, prospective | 300 IS or TIA | To assess the prevalence of asymptomatic >50% coronary artery disease in patients with IS or transient ischemic attack and its relation to vascular risk factors and atherosclerosis. | The prevalence of asymptomatic CAD >50% was 18% (95%CI, 14-23). Asymptomatic CAD was independently associated with risk factors (OR 2.6; 95%CI, 1.0-7.6 for a 10-year risk of coronary disease of <20%; and OR 7.3; 95%CI, 2.8-19.1 for a 10 year-risk of coronary disease > 20%), at least one >50% artery stenosis (OR 4.0; 95%CI, 1.4-11.2), and alcohol consumption (OR 3.1; 95%CI 1.3-7.3). |
| Liao et al.[34] | 2009 | Eur J Neurol | Observational, prospective | 9,180 IS | To assess frequency and clinical outcome (mRS, death) of myocardial infarction following acute IS. | At hospital discharge, 64.9% of IS patients with myocardial infarction died or had severe disability. Mortality at 1 year was 56.4% in IS patients with myocardial infarction and 21.9% in the entire cohort. Myocardial infarction was associated with death or severe dependence at discharge (OR 2.51; 95%CI 1.75-3.59) and mortality within 1 year (HR 1.83; 95%CI 1.51-2.23). |
| Hoshino et al.[74] | 2008 | Intern Med | Observational, prospective | 100 IS | To assess the utility of elements of brain ischemia to predict subclinical CAD in IS. | 36% of patients developed CAD. Patients with subclinical CAD had higher rates of intracranial artery stenosis (78.1% vs 35.1%, p<0.0001) and silent brain infarction (69.4% vs 46.9%, p=0.03). 61% of patients with both brain infarction and intracranial artery stenosis had subclinical CAD. |
| Gongora Rivera et al.[75] | 2007 | Stroke | Observational, prospective | 803 neurologic disease (341 IS) | To investigate the prevalence of coronary atherosclerosis and myocardial infarction after fatal ischemic stroke. | Coronary plaques were found in 72.4%, coronary stenosis in 37.5%, and myocardial infarction in 40.8% of IS patients; and in 26.8%, 10.1%, and 12.8%, respectively, of patients with other neurologic diseases (p<0.001). The prevalence of coronary plaques was 79%, coronary stenosis 42.9%, and myocardial infarction 46.0% in the presence of plaques in extracranial and intracranial brain arteries, versus 50.8%, 17.9%, and 23.9% in the absence of plaques (p<0.01). |
| Prosser et al.[76] | 2007 | Stroke | Observational, prospective | 846 IS | To assess cardiac events after stroke. | Among IS patients, 4.1% died from cardiac causes and 19.0% suffered at least one serious cardiac adverse event. Cardiac mortality was highest after 2 weeks. Risk factors for cardiac adverse events were: history of heart failure (OR 3.33 95%CI 2.28-4.89, p<0.001), diabetes (OR 2.11 95%CI 1.39-3.21, p<0.001), higher baseline creatinine (OR 1.77 95%CI 1.16-2.70, p=0.008), severe IS (OR 1.98 95%CI 1.34-2.91, p=0.001), and prolonged QTc or ventricular extrasystoles (OR 1.93 95%CI 1.31-2.85], p=0.001). |
| Dhamoon et al.[77] | 2006 | Neurology | Observational, prospective | 655 IS | To assess recurrent stroke and fatal cardiac events after IS (death secondary to myocardial infarction, congestive heart failure, sudden death/arrhythmia, and cardiopulmonary arrest). | The risk of recurrent stroke was higher than that of fatal cardiac events. The 5-year risk of recurrent IS was 18.3%, versus 8.6% for myocardial infarction and cardiac events. |
| **Cardiac dysfunction and remodeling** | | | | | | |
| Hashimoto et al.[78] | 2019 | Heart vessels | Observational, prospective | 196 IS | To evaluate left atrial remodeling through echocardiography and its association with recurrent cerebrovascular events. | Lower left atrial remodeling index was associated with recurrent cerebrovascular events (0.50±0.45 vs. 1.10±0.95, p< 0.001). |
| Li et al.[79] | 2019 | J Stroke Cerebrovasc Dis | Observational, prospective | 685 IS | To assess the ability of left ventricular ejection fraction and clinically defined heart failure to predict 90-day functional outcome after ischemic stroke. | Left ventricular ejection fraction was independently associated with 90-day disability (OR 0.98, 95%CI 0.96-0.99, p=0.011). Left ventricular ejection fraction, heart failure, left ventricular dysfunction, and heart failure/left ventricular systolic dysfunction were independently associated with atrial fibrillation (p<0.01 each), with similar predictive ability (AUC = 0.74, 0.74, .73, and 0.75, respectively). |
| Gasiorek et al.[80] | 2019 | Hindawi Dis Mark | Observational, prospective | 101 (65 ESUS, 36 control) | To assess arterial stiffness and left ventricular diastolic dysfunction in patients with ESUS and controls. | No differences in aortic diastolic pressure and systolic pressure between ESUS and control groups. Lower LVEF was found in the ESUS group but within normal ranges (60% vs 63%, p=0.009). ESUS patients had lower E′ (median [IQR], 8.6 cm/s [7.1-10.3] vs 12.5 cm/s (9.6-14); p = 0.0008) and systolic (S′) mitral annular velocities (mean ± SD, 7±1 vs 8±1 cm/s; p=0.03) and a higher E/E′ ratio compared to the control group (median, 7.6 [6.1-8.9] vs 6.0 [5.3-6.9], p=0.0002). IVRT was longer in ESUS patients compared to the control group (113±23 vs 97±30ms; p=0.001). |
| Choi et al.[42] | 2017 | Neurology | Observational, prospective | 4,316 IS | To investigate the role of left ventricular wall motion abnormalities and their impact on stroke recurrence. | IS and stroke recurrence occurred in 7.2% and 5.8% patients, respectively. Left ventricular wall motion abnormalities were associated with any stroke (HR 1.707, 95%CI 1.262-2.310) and with IS (HR 1.709, 95%CI 1.222-2.390). The association remained after correction for covariates (HR 1.747, 95%CI 1.292-2.364 for any stroke; HR 1.704, 95%CI 1.219-2.382 for IS). |
| Kim et al.[43] | 2016 | J Stroke Cerebrovasc Dis | Observational, prospective | 1,554 IS | Left ventricular dysfunction and its association with functional outcomes (by mRS) at 3 months. | Stroke-­related disability at discharge and at 3 months were significantly associated with left ventricular dysfunction. Old age, diabetes mellitus, high NIHSS at admission, stroke mechanism, and left ventricular dysfunction were independent predictors of poor functional outcomes at 3 months. |
| Rojek et al.[81] | 2016 | J Hypertens | Observational, prospective | 216 IS | To stratify outcomes by ejection fraction in IS patients. | Patients with favorable outcome had a higher ejection fraction than those with poorer prognosis (54.3±7.9 vs 49.9±9.8%, p=0.005). Ejection fraction >50% was associated with favorable outcome (OR 3.81, 95%CI 1.18-12.35, p=0.02). |
| Olsen et al.[82] | 2015 | Int J Cardiovasc Imaging | Observational, prospective | 244 IS | To assess echocardiographic measures in IS patients to predict outcome. | Approximately 17% of patients died. Dead patients had impaired systolic and diastolic function (by left ventricular ejection fraction and E/e′ ratio).  A decrease in global e′ increased the risk of dying, with a 13-fold increase for patients in the lowest tertile compared to those in the highest tertile (HR 13.4, 3.2-56.3, p<0.001). |
| Burkot et al.[44] | 2015 | J Card Fail | Observational, prospective | 566 IS | To assess whether decompensated heart failure is an independent predictor of functional outcome (by mRS) after IS. | Decompensated heart failure was diagnosed in 17% of patients. Preserved ejection fraction was present in 57% of patients. After adjusting for age, stroke severity, atrial fibrillation, myocardial infarction, hyperglycemia, pneumonia, fever, leukocytosis, proteinuria, and reduced ejection fraction, decompensated heart failure remained an independent predictor of worse outcome (OR 2.34, 95%CI 1.12-4.89; p=0.02). |
| Ntaios et al.[83] | 2014 | Eur J Neurol | Observational, prospective | 2,730 IS | To investigate cardiovascular outcomes (myocardial infarction, stroke recurrence, angina pectoris, acute heart failure, sudden cardiac death, and aortic aneurysm rupture) and mortality after IS. | Ten-year cardiovascular mortality in IS patients was 46.6% (95%CI 40.6–52.8. Cardiovascular mortality for lacunar IS (22.1%, 95%CI 16.2–28.0) or undetermined IS (35.2%, 95%CI 27.8–42.6) were similar or higher than large-artery atherosclerotic IS (28.7%, 95%CI 22.4–35.0). |
| Seo et al.[84] | 2014 | Stroke | Observational, prospective | 1,589 IS and TIA | To investigate whether left ventricular diastolic dysfunction is an important mechanism for stroke in cryptogenic IS patients. | Severe left ventricular diastolic dysfunction showed similar rate between IS patients with atrial fibrillation or not (p=0.173) but was higher than those without atrial fibrillation (p=0.008). Left ventricular diastolic dysfunction of grade II (OR 4.37; 95%CI 2.99-6.41) and grade III (OR 5.60; 95%CI 3.42-9.17) were independently associated with stroke patients with atrial fibrillation. |
| Ambrosi et al.[85] | 2010 | Int J Cardiol | Observational, prospective | 55 IS | To investigate whether pro-BNP is related to diastolic dysfunction in IS patients. | Left ventricular ejection fraction was evaluated in IS patients. All patients developed diastolic or systolic dysfunctions. |
| Prosser et al.[76] | 2007 | Stroke | Observational, prospective | 846 IS | To identify cardiac risk after stroke and develop a predictive model for serious cardiac adverse events. | Among IS patients, 4.1% died from cardiac causes (especially in the second week after IS), and 19% developed at least one cardiac complication. History of heart failure (OR 3.33, CI 2.28-4.89, p<0.001), diabetes (OR 2.11, CI 1.39-3.21, p<0.001), higher baseline creatinine (OR 1.77, CI 1.16-2.70, p=0.008), severe stroke (OR 1.98, CI 1.34-2.91, p=0.001), and prolonged QTc or ventricular extrasystoles (OR 1.93, CI 1.31-2.85, p=0.001) were identified as predictors of serious cardiac events. |

*IS, ischemic stroke, HS, hemorrhagic stroke; ICH, intracerebral hemorrhage; SAH, subarachnoid hemorrhage; RCT, randomized controlled trial; vs, versus; aOR, adjusted odds ratio; CI, confidence interval; mRS, modified Rankin scale; AF, atrial fibrillation; HR, hazard ratio; RR, relative risk; ESUS, embolic stroke of undetermined source; LVEF, left ventricular ejection fraction; E′, mean early diastolic velocity; IVRT, isovolumic relaxation time; SD, standard deviation; IQR, interquartile range. CAD, coronary artery disease; TIA, transient ischemic attack; NIHSS, National Institutes of Health Stroke Scale*

**Table S3**. **Blood pressure control in ischemic stroke and its association with outcome**

Studies are presented from most to least recent; Only observational and randomized studies were included.

| **Blood pressure and outcome** | | | | | | | |
| --- | --- | --- | --- | --- | --- | --- | --- |
| **Author** | **Year** | **Journal** | **Type of study** | **Number of patients included** | **Timing after stroke** | **Outcomes** | **Results** |
| Verschoof et al.[86] | 2019 | Stroke | Observational, prospective | 2,124 IS | After IS, acute (hours) | To compare the effect of baseline systolic blood pressure after stroke on outcome (in-hospital mortality, major complications <7 days of stroke onset, and functional outcome at 90 days calculated by mRS); low systolic blood pressure defined as less than 10^th^ percentile vs normal systolic blood pressure ≥ 10^th^ percentile. | In-hospital mortality was 8.0% in patients with low systolic blood pressure vs 4.2% in those with normal systolic blood pressure (aOR 1.58; 95%CI, 1.13-2.21). Complications (16.0% vs 6.5%; aOR, 2.56; 95%CI, 1.60-4.10), heart failure (2.4% vs 0.1%; aOR, 17.85; 95%CI, 3.36-94.86), and functional outcome at 90 days did not differ (aOR 1.24; 95%CI, 0.95-1.61). |
| De Havenon et al.[87] | 2019 | JRSM Cardiovasc Dis | Secondary analysis of RCT | 17,896 IS | After IS, chronic (years) | To assess if BP variability has an effect on recurrent stroke (ischemic or hemorrhagic stroke both), major cardiovascular events (death from cardiovascular causes, recurrent stroke, myocardial infarction, or new or worsening heart failure), and all-cause mortality. | Both systolic and diastolic blood pressure variability were associated with recurrent stroke (total incidence of recurrent stroke 4.2%, of which 3.6% IS), major cardiovascular events, and all-cause mortality, but significant only for IS. For every 10-point increase in BP variability, the HR for recurrent ischemic stroke was 1.15 (95%CI, 1.02-1.32; p=0.02), for major cardiovascular events 1.19 (95%CI, 1.09-1.31; p<0.001), and for all-cause mortality 1.24 (95%CI, 1.10-1.39; *P*<0.001). |
| Lee et al.[88] | 2018 | Stroke | Observational, prospective | 9,840 IS | After IS, acute (within 48 hours) | To assess the influence of pulse pressure on outcome at 1-year follow-up after IS. | At 1-year follow-up, 4.3% of patients had experienced recurrent stroke, 0.2% myocardial infarction, and 7.3% had died (7.3%). The overall effects of pulse pressure and blood pressure parameters outcomes were significant (*p*<0.05). Pulse pressure was a stronger predictor of outcome than blood pressure. |
| Kang et al.[89] | 2017 | Plos One | Observational, prospective | 2,545 IS | After IS, acute (within 24 hours) | To investigate if blood pressure variability is related to outcome (early neurological deterioration). | Systolic blood pressure variability, evaluated daily, was associated with neurological deterioration independent of blood pressure variability on the previous day. Mean blood pressure variability on day 2 (aOR, 1.08; 95%CI, 1.03–1.13) and day 3 were independently associated with onset of early neurological deterioration (aOR 1.07, 95%CI 1.01–1.14). |
| Bangalore et al.[90] | 2017 | Eur Heart J | Observational, prospective | 309,611 IS | After IS, acute (within 24 hours) | To assess the relationship between lower and higher systolic blood pressure and functional outcome or hemorrhagic complications. | Both lower (120-150 mmHg) and higher (150-200 mmHg) systolic blood pressure were associated with higher risk of in-hospital death (OR 1.16, 95%CI 1.13-1.20 for lower; OR 1.15, 95%CI 1.12-1.18 for higher). Risk of hemorrhagic complications after thrombolytic therapy was lower for lower systolic blood pressure (OR 0.89 95%CI 0.83-0.97) and increased for higher systolic blood pressure (OR 1.21 95%CI 1.11-1.32). |
| Lee et al. [91] | 2015 | Stroke | Meta-analysis | 12,703 IS from 13 RCTs | After IS, acute (within 72 hours) | To evaluate unfavorable outcomes at 3 months or at trial endpoint (dependency or death). Meta-analysis restricted to RCTs which assesses blood pressure lowering versus control within 72 hours. | Blood-pressure lowering in the acute phase of IS did not change the risk of death or dependency at 3 months (RR, 1.04; 95%CI, 0.96-1.13; p=0.35). |
| Zhao et al.[92] | 2015 | Medicine | Meta-analysis | 5,672 IS, 5,416 controls | After IS, acute | To evaluate whether lowering blood pressure during the acute phase of ischemic stroke improves outcomes. | Treated patients showed a greater decrease in blood pressure than controls. Short-term and long-term outcomes did not differ (OR 1.041, 95%CI 0.936-1.159, p= 0.457; OR 1.013, 95%CI 0.915-1.120, p = 0.806, respectively). Short- and long-term mortality was similar between the two groups (OR 1.020, 95%CI 0.749-1.388, p =0 .902; OR 1.039, 95%CI 0.883-1.222, p = 0.644, respectively). |
| Manning et al. [93] | 2015 | Stroke | Meta-analysis | 1,359 IS | After IS, acute | To assess whether blood pressure variability is associated with outcome. | Systolic blood pressure variability was significantly associated with poor functional outcome (OR 1.2; 95%CI 1.1-1.3). |
| Oh et al.[94] | 2015 | Int J Stroke | RCT | 393 IS (valsartan 195, control 198) | After IS, acute (within 7 days) | To assess the efficacy and safety of blood pressure reduction within 48 hours after IS and its impact on outcome (death or dependency by mRS at 90 days). | Blood pressure reduction with valsartan was not associated with outcome improvement. In fact, 24.6% of IS patients in the valsartan group showed dependency vs 22.6% in the control group (OR 1.11; 95%CI, 0.69–1.79; p=0.667). The incidence of major vascular events did not differ between groups (OR 1.41; 95%CI, 0.44–4.49; p=0.771). The valsartan group showed increased early neurological deterioration (OR 2.43; 95%CI, 1.25–4.73; p=0.008). |
| He et al.[95] | 2014 | JAMA | RCT | 4,071 (2038 antihypertensive, 2033 control) | After IS, acute (within days) | Primary outcome: death and major disability (by mRS) at 14 days (aim of anti-hypertensive treatment: lowering systolic blood pressure by 10-25% within 24 hours, achieving blood pressure less than 140/90 mmHg within 7 days, and maintaining this level during hospitalization; aim of control: to discontinue antihypertensive treatment during hospitalization). | Mean systolic blood pressure was reduced in the antihypertensive group and in the control group within 24 hours (p<0.001). Mean systolic blood pressure was 137.3 mmHg in the antihypertensive group and 146.5 mmHg in the control group 7 days after randomization (p<0.001). Primary outcome did not differ between groups (OR 1.00, 95%CI, 0.88-1.14, p=0.98) at 14 days. The secondary outcome (death and major disability at 3 months) did not differ between groups (OR 0.99, 95%CI, 0.86-1.15; p=0.93). |
| Sandeset et al.[96]blood pressure jama | 2012 | Stroke | Observational, prospective | 2,029 IS | After IS, acute (within 30 hours) | To investigate the effect of blood pressure changes during the first 2 days of stroke on the risk of adverse events and poor outcome. | Larger changes or no change in systolic blood pressure increased the risk of early adverse events vs a smaller decrease in pressure (OR 2.08; 95%CI, 1.19-3.65 and OR 1.96; 95%CI, 1.13-3.38). IS patients with increase/no change in systolic blood pressure showed increased risk of poor neurological outcome (p=0.001). No differences were found in functional outcome at 6 months. |
| Aries et al.[97] | 2012 | Stroke | Observational, prospective | 177 IS | After IS, acute (within 3 days) | To assess the relationship between early upright position and blood pressure and functional outcome. | About 60% of patients were able to stand. The authors found elevated blood pressure (p<0.001) during early upright positioning in patients with acute IS, which was independently associated with favorable outcome (p<0.003). |
| Geeganage et al.[98] | 2011 | Stroke | Observational, prospective | 1,479 IS | After IS, acute (within hours) | To assess the relationship between baseline blood pressure, heart rate, and other hemodynamic parameters, and outcomes at 10 days (death, deterioration). | Systolic blood pressure (aOR, 1.02; 95%CI, 1.01–1.03), mean arterial pressure (OR, 1.02; 95%CI, 1.01–1.04), pulse pressure (OR, 1.02; 95%CI, 1.01–1.03), and blood pressure variability (OR, 1.03; 95%CI, 1.01–1.05) were independently associated with death, neurological deterioration, deterioration and recurrent stroke. |
| Tomii et al.[99] | 2011 | Stroke | Observational, prospective | 104 IS | After IS, acute (within 7 days) | To assess the effect of blood pressure and heart rate on outcome measure (mRS) after IS. | A total of 66% patients achieved independence, of whom 11% had poor outcomes. Mean heart rate, mean blood pressure, mean systolic blood pressure, and diastolic blood pressure were positively associated with poor outcome at day 1 and day 7. |
| Sandset et al.[100] | 2011 | Lancet | RCT | 2,029 (1017 candesartan, 1012 placebo) | After IS, acute (within 7 days) | To assess vascular death, myocardial infarction, and stroke during the first 6 months, as well as functional outcome at 6 months (by mRS). | Blood pressures were lower for the candesartan group vs placebo (p<0.0001). Outcomes at 6 months did not differ between treatment groups (HR 1·09, 95%CI 0·84-1·41; p=0.52). Higher risk of poor outcome was found in the candesartan group (aOR 1.17, 95%CI 1.00-1.38; p=0.048). Thus, the evidence suggested a harmful effect of lower blood pressure. |
| Sare et al.[101] | 2009 | Stroke | Observational, prospective | 1,722 IS | After IS, acute (within 8 hours) | To assess neurological status at 7 days (by NIHSS), and functional outcome at 90 days (by mRS), in respect to blood pressure variability after IS. | High systolic blood pressure was associated with neurological impairment (OR 1.06, 95CI 1.01-1.12) and poor functional outcome. Smaller vs larger declines in systolic blood pressure within 24 hours were associated with poor NIHSS (OR 1.16, 95%CI 1.05-1.27) and functional outcome (OR 1.23, 95%CI 1.13-1.34). |
| Bath et al.[102] | 2009 | Stroke | RCT | 1,360 IS (647 telmisartan, 713 placebo) | After IS, acute (within 72 hours) | To assess functional outcome at 30 days and secondary outcomes (death, dependency by mRS, stroke recurrence, and hemodynamic measures) at up to 90 days. | For mRS (OR 1.03; 95%CI 0.84-1.26; p=0.81), death (OR 1.05; 95%CI 0.27-4.04), and stroke recurrence (OR 1.40; 95%CI, 0.68-2.89; p=0.36), there were no differences between groups. Telmisartan lowered blood pressure (p<0.001), pulse pressure (p<0.002), and rate-pressure product (p=0.0004) vs placebo. |
| Potter et al.[103] | 2009 | Lancet Neurol | RCT | 179 IS (58 labetalol, 58 lisinopril, 63 placebo) | After IS, acute (within 36 hours) | To assess the relationships between blood pressure management and outcome (death or dependency at 2 weeks). | Death or dependency at 2 weeks occurred in 61% of the intervention group and 59% of the placebo group (RR 1.03, 95%CI 0.80-1.33; p=0.82). Active treatment was not associated with early neurological deterioration (RR 1.22, 0.33-4.54; p=0.76), but was associated with greater fall in systolic blood pressure within the first 24 hours in intervention vs placebo (p=0.004). No serious adverse events were reported with antihypertensive treatment (RR 0.91, 0.69-1.12; p=0.50), and 3-month mortality was reduced (9.7% vs 20.3%, HR 0.40, 95%CI 0.2-1.0; p=0.05). |
| Sare et al.[101] | 2009 | Stroke | Observational, prospective | 1,489 IS | After IS, acute (within 48 hours) | To assess the relationships between baseline blood pressure and computed tomography findings. OR was calculated for each 10 mmHg of change in blood pressure. | Higher systolic blood pressure was associated with abnormal computed tomography (OR 1.12; 95%CI, 1.05–1.17) and old infarction (OR 1.12; 95%CI, 1.06–1.17) at baseline, and visible infarction after 10 days (OR 1.06; 95%CI, 1.00–1.13). A lower systolic blood pressure was associated with signs of acute infarction (OR, 0.94; 95%CI, 0.89–0.99). |
| Eveson et al.[104] | 2007 | Am J Hypertens | RCT | 40 IS | After IS, acute (within 24 hours) | To compare the effect of early lisinopril vs placebo on pressure control. | Of 40 patients, 18 received lisinopril and 22 received placebo. Systolic and diastolic blood pressure differed significantly between the lisinopril and placebo groups (p<0.05 and p<0.07, respectively). Systolic pressure, mean arterial pressure, diastolic pressure, and pulse pressure were significantly lower in the lisinopril group than in the placebo group at day 14 (p<0.01). Neurological outcome did not differ. |
| Rodriguez-Garcia et al.[105] | 2004 | Am J Hypertens | Observational, prospective | 434 IS | After IS, acute (within 24 hours) | To characterize blood pressure in acute ischemic stroke and to determine its relationship with short-term functional outcome (mRS 4 to 6 on day 7 or death during hospital stay). | Patients with non-lacunar stroke showed significantly higher NIHSS scores, systolic blood pressure, and heart rate than lacunar stroke patients. Death or dependency was associated with NIHSS score (OR 1.08 95%CI 1.04-1.13), 24-h systolic blood pressure (OR 2.35, 95%CI 1.10-5.52), and plasma glucose >125 mg/dL on admission (OR 1.88, 95%CI 1.03-3.57), whereas a decrease in systolic blood pressure on day 7 was associated with lower death or dependency rate (OR 0.46, 95%CI 0.24-0.88). |
| Aslanyan et al.[106] | 2004 | Stroke | Secondary analysis of RCT | 1,455 IS | After IS, acute (within 60 hours) | To evaluate if elevated pulse pressure (difference between systolic BP and diastolic BP) is independently associated with poor stroke outcome at 3 months. | Elevated weighted average PP during the first 60 hours was associated with poor outcome by mortality (OR 1.18 95%CI 1.11–1.26), Barthel index (OR 1.28 95%CI 1.21–1.36), NIHSS (OR 1.22 95%CI 1.13–1.31), and mRS (OR 1.23 95%CI 1.14–1.32). Elevated baseline PP was associated with Barthel index and Rankin score. |
| Schrader et al.[107] | 2003 | Stroke | RCT | 339 IS | After IS, | To assess the efficacy of candesartan on lowering blood pressure in IS patients and its effects on outcome. | 12-month mortality and number of vascular events differed in favor of the treatment group (OR 0.475; 95%CI, 0.252-0.895). The trial was interrupted prematurely. |
| Vlcek et al.[108] | 2003 | Ann Emerg Med | Observational, prospective | 372 IS | After IS, acute (within 24 hours) | To evaluate the relation between blood pressure variability and early neurologic outcome (at day 5 of admission with mRS). | This study showed a significant association only for diastolic blood pressure less than 25% from admission until 24 hours after admission, which was associated with a 3.8-fold increase in aOR (95%CI 1.2-12.1) for poor neurologic outcome on day 5. |
| Horn et al.[109] | 2001 | Stroke | RCT | 454 IS (225 nimodipine, 29 placebo) | After IS, acute (within 6 hours) | To assess poor outcome (death or dependency after 3 months), neurological status and blood pressure (after 24 hours), mortality (after 10 days), and adverse events between groups. | Nimodipine had no effect. After 3 months, 32% of patients in the nimodipine group had a poor outcome vs 27% in the placebo group (RR 1.2; 95%CI, 0.9-1.6). |
| Ahmed et al.[110] | 2000 | Stroke | RCT | 265 IS (80 placebo, low-dose nimodipine 92, high-dose nimodipine 93) | After IS, acute (within 24 hours) | To assess the correlation between blood pressure changes within 2 days and outcome at 21 days. | No correlation was found between pressure changes and outcome. Nimodipine reduced systolic and diastolic blood pressure from baseline compared with placebo during the first few days. In the high-dose group (beta=0.49, p=0.048), diastolic blood pressure reduction and worsening of neurological score were found. Patients with a diastolic blood pressure reduction ≥20% (high-dose group) had a much higher risk of death or dependency (OR 10.16, 95%CI 1.02-101.74) and of death alone (OR 4.34, 95%CI 1.13-16.62) compared with placebo. |
| Kaste et al.[111] | 1994 | Stroke | RCT | 350 IS | After IS, acute | To assess whether nimodipine can improve functional outcome after IS (mRS, mobility and neurological score). | There was no difference in outcome between nimodipine and placebo. However, at 1 month and 3 months, the case-fatality rate was higher in the nimodipine group than in the placebo group (p=0.004 and p=0.030, respectively). During the first 7 days nimodipine lowered both systolic (p=0.005) and diastolic (p=0.013) blood pressure. |

*IS, ischemic stroke, HS, hemorrhagic stroke; RCT, randomized controlled trial; vs, versus; aOR, adjusted odds ratio; CI, confidence interval; mRS, modified Rankin scale; AF, atrial fibrillation; HR, hazard ratio; RR, relative risk; mean ± SD, mean and standard deviation; median (IQR), median and interquartile range; NIHSS, National Institute of Health Stroke Scale*

**Table S4**. **Troponin T as acute biomarker of cardiovascular complications after ischemic stroke**

| **Author** | **Year** | **Journal** | **Type of study** | **Number of patients** | **Type of cardiac dysfunction** | **Timing after stroke** | **Primary endpoint** | **Results** |
| --- | --- | --- | --- | --- | --- | --- | --- | --- |
| Csecsei et al.[112] | 2018 | J Stroke Cerebrovasc Dis | Observational, prospective | 35 IS | Troponin T | After IS, acute (6-24 hours) | To assess the ability of cardiac troponin to predict outcome after acute IS. | Serum cardiac troponin T at 24 hours > 9.4 predicted worse NIHSS on discharge (p=0.002, sensitivity 81%, specificity 74%). Troponin T at 6 and 24 hours was higher in non-survivors compared with survivors (p=0.03). Troponin T at 24 hours was an independent predictor of worse NIHSS at discharge (OR 1.58, 95%CI 1.063-2.370, p=0.024). |
| Batal et al.[113] | 2016 | J Crit Care | Observational, prospective | 1,718 IS | Troponin I | After IS, acute (hours) | To assess in-hospital and long-term mortality in IS patients. | Patients with MI (39%) had the highest mortality (p=0.001) and the lowest survival rate (p=0.0001). Peak troponin I was independently associated with long-term mortality (p=0.0001). |
| Beaulieau-Boire et al.[114] | 2013 | J Stroke Cerebrovasc Dis | Observational, prospective | 408 IS | Troponin I | After IS, acute (within 7 days) | To evaluate new-onset AF (by Holter within 1 week of admission) in patients without previous AF; to assess impact of new-onset AF on Holter, death, myocardial infarction, and stroke within 3 months. | A total of 11.3% patients showed high troponin I. Elevated troponin I levels predicted a composite outcome of stroke, myocardial infarction, and death at 3 months (50.0% vs 16.1%; p=0.0001). |
| He et al.[115] | 2018 | BMC Neurol | Observational, prospective | 570 IS | Troponin T | After IS, sub-acute (3 months) | To assess troponin T values and their association with outcome (mRS) at 3 months. | Serum troponin T was elevated in 22.87% of patients, especially in patients with previous lacunar stroke (aOR 2.84, 95%CI 1.48-4.7, p=0.001). Significant associations were found between serum troponin T and death (aOR 3.14, 95%CI 1.04-4.51, p=0.02), major disability (aOR 2.07, 95%CI 1.04-4.51, p=0.04), and the composite outcome (aOR 2.22, 95%CI 1.10-4.48, p=0.03). |
| Peddada et al.[116] | 2016 | Cerebrovasc Dis | Observational, prospective | 1,145 IS | Troponin | After IS | To assess whether troponin elevation after IS is associated with hospital mortality. | 17% of IS patients had elevated troponin. Patients with elevated troponin levels had higher hospital mortality (27 vs 8%, p<0.001) for all categories of death (neurologic, 17 vs 7%; cardiac, 5 vs <1%; other, 5 vs <1%). |
| Maoz et al.[117] | 2015 | J Mol Neurosci | Observational, prospective | 212 IS | Troponin | After IS | To investigate the association between increased serum troponin, stroke severity, and mortality. | A total of 16.51% of patients had increased troponin. Troponin level was associated with older age (p<0.001), poor kidney function (p<0.001), and known ischemic heart disease (p=0.049). Patients with higher troponin showed increased stroke severity by NIHSS (p<0.001). |
| Fure et al.[118] | 2006 | J Intern Med | Observational, prospective | 279 IS | Troponin T | After IS, acute (within 24 hours) | To assess ECG changes and their association with troponin elevation and outcome after IS. | The most common ECG changes were prolonged QTc (36.0%), ST depression (24.5%), atrial fibrillation (19.9%) and T wave inversion (17.8%). ST depression and Q waves were associated with increased troponin T, which was elevated in 9.6% of patients. Increased troponin T was significantly associated with a poor short-term outcome (mRS). |

*IS, ischemic stroke; AF, atrial fibrillation; MI, myocardial infarction; sens, sensitivity; spec, specificity; NIHSS, National Institute of Health Stroke Scale; ECG, electrocardiogram; OR, odds ratio; aOR, adjusted odds ratio; mRS, modified Ranking scale*

**REFERENCES**

1. Xiong L, Tian G, Leung H, Soo YOY, Chen X, Ip VHL, et al. Autonomic dysfunction predicts clinical outcomes after acute ischemic stroke: A prospective observational study. Stroke. 2018;49:215–8.

2. Nayani S, Sreedharan SE, Namboodiri N, Sarma PS, Sylaja PN. Autonomic dysfunction in first ever ischemic stroke: Prevalence, predictors and short term neurovascular outcome. Clin Neurol Neurosurg. 2016;150:54–8.

3. Idiaquez J, Farias H, Torres F, Vega J, Low DA. Autonomic symptoms in hypertensive patients with post-acute minor ischemic stroke. Clin Neurol Neurosurg. 2015;139:188–91.

4. Xiong L, Leung H, Chen XY, Han JH, Leung T, Soo Y, et al. Preliminary findings of the effects of autonomic dysfunction on functional outcome after acute ischemic stroke. Clin Neurol Neurosurg. 2012;114:316–20.

5. Diserens K, Vuadens P, Michel P, Reichhart M, Herrmann FR, Arnold P, et al. Acute autonomic dysfunction contralateral to acute strokes: a prospective study of 100 consecutive cases. Eur J Neurol. 2006;13:1245–50.

6. Tanislav C, Kostev K. Late Detection of Atrial Fibrillation after Stroke: Implications for the Secondary Prevention. Eur Neurol. 2019;81:262–9.

7. Christensen H, Boysen G, Christensen AF, Johannesen HH. Insular lesions, ECG abnormalities, and in outcome in acute stroke. J Neurol Neurosurg Psychiatry. 2005;76:269–71.

8. Wachter R, Gröschel K, Gelbrich G, Hamann GF, Kermer P, Liman J, et al. Holter-electrocardiogram-monitoring in patients with acute ischaemic stroke (Find-AFRANDOMISED): an open-label randomised controlled trial. Lancet Neurol. 2017;16:282–90.

9. Adeoye AM, Ogah OS, Ovbiagele B, Akinyemi R, Shidali V, Agyekum F, et al. Prevalence and Prognostic Features of ECG Abnormalities in Acute Stroke: Findings From the SIREN Study Among Africans. Glob Heart. 2017;12:99–105.

10. Hromádka M, Seidlerová J, Rohan V, Baxa J, Šedivý J, Rajdl D, et al. Prolonged Corrected QT Interval as a Predictor of Clinical Outcome in Acute Ischemic Stroke. J Stroke Cerebrovasc Dis. 2016;25:2911–7.

11. Fernández-Menéndez S, García-Santiago R, Vega-Primo A, González Nafría N, Lara-Lezama LB, Redondo-Robles L, et al. Arritmias cardiacas en la unidad de ictus: Análisis de los datos de la monitorización cardiaca. Neurologia. 2016;31:289–95.

12. Yayehd K, Irles D, Akret C, Vadot W, Rodier G, Berremili T, et al. Detection of paroxysmal atrial fibrillation by prolonged electrocardiographic recording after ischaemic stroke in patients aged &lt; 60 years: A study with 21-day recording using the SpiderFlash® monitor. Arch Cardiovasc Dis. 2015;108:189–96.

13. Bobinger T, Kallmünzer B, Kopp M, Kurka N, Arnold M, Hilz MJ, et al. Prevalence and impact on outcome of electrocardiographic early repolarization patterns among stroke patients: a prospective observational study. Clin Res Cardiol. 2015;104:666–71.

14. Purushothaman S, Salmani D, Prarthana KG, Bandelkar SMG, Varghese S. Study of ECG changes and its relation to mortality in cases of cerebrovascular accidents. J Nat Sci Biol Med. 2014;5:434–6.

15. González Toledo ME, Klein FR, Riccio PM, Cassará FP, Muñoz Giacomelli F, Racosta JM, et al. Atrial fibrillation detected after acute ischemic stroke: Evidence supporting the neurogenic hypothesis. J Stroke Cerebrovasc Dis. 2013;22.

16. Kallmünzer B, Breuer L, Kahl N, Bobinger T, Raaz-Schrauder D, Huttner HB, et al. Serious cardiac arrhythmias after stroke: incidence, time course, and predictors--a systematic, prospective analysis. Stroke. 2012;43:2892–7.

17. Ritter MA, Rohde A, Heuschmann PU, Dziewas R, Stypmann J, Nabavi DG, et al. Heart rate monitoring on the stroke unit. What does heart beat tell about prognosis? An observational study. BMC Neurol. 2011;11.

18. Christensen H, Fogh Christensen A, Boysen G. Abnormalities on ECG and telemetry predict stroke outcome at 3 months. J Neurol Sci. 2005;234:99–103.

19. Doğan A, Tunç E, Oztürk M, Erdemoğlu AK. [Comparison of electrocardiographic abnormalities in patients with ischemic and hemorrhagic stroke]. Anadolu Kardiyol Derg. 2004;4:135–40.

20. Li C, Dong W. [Abnormal dynamic electrocardiogram in patients with acute cerebral infarction]. Zhonghua nei ke za zhi. 1999;38:239–41.

21. Bhatia R, Sharma G, Patel C, Garg A, Roy A, Bali P, et al. Coronary Artery Disease in Patients with Ischemic Stroke and TIA. J Stroke Cerebrovasc Dis. 2019;:104400.

22. Alqahtani F, Aljohani S, Tarabishy A, Busu T, Adcock A, Alkhouli M. Incidence and outcomes of myocardial infarction in patients admitted with acute ischemic stroke. Stroke. 2017;48:2931–8.

23. Mathias TL, Albright KC, Boehme AK, Monlezun D, George AJ, Jones E, et al. The Impact of Myocardial Infarction vs. Pneumonia on Outcome in Acute Ischemic Stroke. J Cardiovasc Dis. 2014;2:1–3.

24. Gattringer T, Niederkorn K, Seyfang L, Seifert-Held T, Simmet N, Ferrari J, et al. Myocardial infarction as a complication in acute stroke: Results from the austrian stroke unit registry. Cerebrovasc Dis. 2014;37:147–52.

25. Cha MJ, Lee HS, Kim YD, Nam HS, Heo JH. The association between asymptomatic coronary artery disease and CHADS2 and CHA2DS2-VASc scores in patients with stroke. Eur J Neurol. 2013;20:1256–63.

26. Kim SJ, Choe YH, Park SJ, Kim GM, Chung CS, Lee KH, et al. Routine cardiac evaluation in patients with ischaemic stroke and absence of known atrial fibrillation or coronary heart disease: Transthoracic echocardiography vs. multidetector cardiac computed tomography. Eur J Neurol. 2012;19:317–23.

27. Micheli S, Agnelli G, Caso V, Alberti A, Palmerini F, Venti M, et al. Acute myocardial infarction and heart failure in acute stroke patients: Frequency and influence on clinical outcome. J Neurol. 2012;259:106–10.

28. Jensen JK, Medina HM, Nørgaard BL, Ovrehus KA, Jensen JM, Nielsen LH, et al. Association of ischemic stroke to coronary artery disease using computed tomography coronary angiography. Int J Cardiol. 2012;160:171–4.

29. Amarenco P, Lavallée PC, Labreuche J, Ducrocq G, Juliard JM, Feldman L, et al. Prevalence of coronary atherosclerosis in patients with cerebral infarction. Stroke. 2011;42:22–9.

30. Cho HJ, Lee JH, Kim YJ, Moon Y, Ko SM, Kim HY. Comprehensive evaluation of coronary artery disease and aortic atherosclerosis in acute ischemic stroke patients: usefulness based on Framingham risk score and stroke subtype. Cerebrovasc Dis. 2011;31:592–600.

31. Yoon YE, Chang HJ, Cho I, Jeon KH, Chun EJ, Choi SI, et al. Incidence of subclinical coronary atherosclerosis in patients with suspected embolic stroke using cardiac computed tomography. Int J Cardiovasc Imaging. 2011;27:1035–44.

32. Calvet D, Touzé E, Varenne O, Sablayrolles JL, Weber S, Mas JL. Prevalence of asymptomatic coronary artery disease in ischemic stroke patients: The precoris study. Circulation. 2010;121:1623–9.

33. Arauz A, Calleja J, Vallejo E, Quintero L. Prevalence of silent myocardial ischemia in single and multiple lacunar infarcts and large vessel disease stroke. Clin Neurol Neurosurg. 2010;112:658–61.

34. Liao J, O’Donnell MJ, Silver FL, Thiruchelvam D, Saposnik G, Fang J, et al. In-hospital myocardial infarction following acute ischaemic stroke: An observational study. Eur J Neurol. 2009;16:1035–40.

35. Hoshino A, Nakamura T, Enomoto S, Kawahito H, Kurata H, Nakahara Y, et al. Clinical utility of evaluating intracranial artery stenosis and silent brain infarction to predict the presence of subclinical coronary artery disease in ischemic stroke patients. Intern Med. 2008;47:1775–81.

36. Lee SJ, Lee KS, Kim YI, An JY, Kim W, Kim JS. Clinical features of patients with a myocardial infarction during acute management of an ischemic stroke. Neurocrit Care. 2008;9:332–7.

37. Seo WK, Yong HS, Koh SB, Suh SI, Kim JH, Yu SW, et al. Correlation of coronary artery atherosclerosis with atherosclerosis of the intracranial cerebral artery and the extracranial carotid artery. Eur Neurol. 2008;59:292–8.

38. Nighoghossian N, Cakmak S, Derex L, Barthelet M, Thibault H, Finet G, et al. Silent coronaropathy: Usefulness of dobutamine stress echocardiography in ischemic stroke. Eur Neurol. 2006;56:211–6.

39. Leys D, Woimant F, Ferrières J, Bauters C, Touboul PJ, Guérillot M, et al. Detection and management of associated atherothrombotic locations in patients with a recent atherothrombotic ischemic stroke: Results of the DETECT survey. Cerebrovasc Dis. 2006;21:60–6.

40. Chimowitz MI, Poole RM, Starling MR, Schwaiger M, Gross MD. Frequency and severity of asymptomatic coronary disease in patients with different causes of stroke. Stroke. 1997;28:941–5.

41. Di Pasquale G, Pinelli G, Grazi P, Andreoli A, Corbelli C, Manini GL, et al. Incidence of silent myocardial ischaemia in patients with cerebral ischaemia. Eur Heart J. 1988;9 suppl N:104–7.

42. Choi JY, Cha J, Jung JM, Seo WK, Oh K, Cho KH, et al. Left ventricular wall motion abnormalities are associated with stroke recurrence. Neurology. 2017;88:586–94.

43. Kim WJ, Nah HW, Kim DH, Cha JK. Association between Left Ventricular Dysfunction and Functional Outcomes at Three Months in Acute Ischemic Stroke. J Stroke Cerebrovasc Dis. 2016;25:2247–52.

44. Burkot J, Kopec G, Pera J, Slowik A, Dziedzic T. Decompensated Heart Failure is a Strong Independent Predictor of Functional Outcome after Ischemic Stroke. J Card Fail. 2015;21:642–6.

45. Verma AK, Aarotale PN, Dehkordi P, Lou JS, Tavakolian K. Relationship between ischemic stroke and pulse rate variability as a surrogate of heart rate variability. Brain Sci. 2019;9.

46. Tobaldini E, Toschi-Dias E, Appratto de Souza L, Rabello Casali K, Vicenzi M, Sandrone G, et al. Cardiac and Peripheral Autonomic Responses to Orthostatic Stress During Transcutaneous Vagus Nerve Stimulation in Healthy Subjects. J Clin Med. 2019;8:496.

47. Grilletti JVF, Scapini KB, Bernardes N, Spadari J, Bigongiari A, Mazuchi FS, et al. Impaired baroreflex sensitivity and increased systolic blood pressure variability in chronic post-ischemic stroke. Clinics. 2018;8:e253.

48. He L, Wang J, Zhang L, Zhang X, Dong W, Yang H. Decreased fractal dimension of heart rate variability is associated with early neurological deterioration and recurrent ischemic stroke after acute ischemic stroke. J Neurol Sci. 2019;396:42–7.

49. Nozoe M, Yamamoto M, Kobayashi M, Kanai M, Kubo H, Shimada S, et al. Heart Rate Variability during Early Mobilization in Patients with Acute Ischemic Stroke. Eur Neurol. 2018;80:19–27.

50. Ha SY, Park KM, Park J, Kim SE, Lee BI, Shin KJ. Autonomic function test in progressive lacunar infarction. Acta Neurol Scand. 2018;138:32–40.

51. Rodriguez J, Blaber AP, Kneihsl M, Trozic I, Ruedl R, Green DA, et al. Poststroke alterations in heart rate variability during orthostatic challenge. Medicine (Baltimore). 2017;96.

52. Xu YH, Wang XD, Yang JJ, Zhou L, Pan YC. Changes of deceleration and acceleration capacity of heart rate in patients with acute hemispheric ischemic stroke. Clin Interv Aging. 2016;11:293–8.

53. Constantinescu V, Matei D, Cuciureanu D, Corciova C, Ignat B, Popescu CD. Cortical modulation of cardiac autonomic activity in ischemic stroke patients. Acta Neurol Belg. 2016;116:473–80.

54. Erdur H, Scheitz JF, Grittner U, Laufs U, Endres M, Nolte CH. Heart rate on admission independently predicts in-hospital mortality in acute ischemic stroke patients. Int J Cardiol. 2014;176:206–10.

55. Graff B, Ga̧secki D, Rojek A, Boutouyrie P, Nyka W, Laurent S, et al. Heart rate variability and functional outcome in ischemic stroke: A multiparameter approach. J Hypertens. 2013;31:1629–36.

56. Xiong L, Leung HHW, Chen XY, Han JH, Leung TWH, Soo YOY, et al. Comprehensive assessment for autonomic dysfunction in different phases after ischemic stroke. Int J stroke. 2013;8:645–51.

57. Gasecki D, Rojek A, Kwarciany M, Kowalczyk K, Boutouyrie P, Nyka W, et al. Pulse wave velocity is associated with early clinical outcome after ischemic stroke. Atherosclerosis. 2012;225:348–52.

58. Chen CF, Lai CL, Lin HF, Liou LM, Lin RT. Reappraisal of heart rate variability in acute ischemic stroke. Kaohsiung J Med Sci. 2011;27:215–21.

59. Bassi A, Colivicchi F, Santini M, Caltagirone C. Cardiac autonomic dysfunction and functional outcome after ischaemic stroke. Eur J Neurol. 2007;14:917–22.

60. Meyer S, Strittmatter M, Fischer C, Georg T, Schmitz B. Lateralization in autononic dysfunction in ischemic stroke involving the insular cortex. Neuroreport. 2004;15:357–61.

61. Strittmatter M, Meyer S, Fischer C, Georg T, Schmitz B. Location-dependent patterns in cardio-autonomic dysfunction in ischaemic stroke. Eur Neurol. 2003;50:30–8.

62. Sander D, Winbeck K, Klingelhöfer J, Etgen T, Conrad B. Prognostic relevance of pathological sympathetic activation after acute thromboembolic stroke. Neurology. 2001;57:833–8.

63. Korpelainen JT, Huikuri H V., Sotaniemi KA, Myllylä V V. Abnormal heart rate variability reflecting autonomic dysfunction in brainstem infarction. Acta Neurol Scand. 1996;94:337–42.

64. Krawczyk M, Fridman S, Cheng Y, Fang J, Saposnik G, Sposato LA. Atrial fibrillation diagnosed after stroke and dementia risk: cohort study of first-ever ischaemic stroke patients aged 65 or older. EP Eur. 2019; pii:euz237.

65. Hsieh CY, Lee CH, Wu DP, Sung SF. Characteristics and outcomes of ischemic stroke in patients with known atrial fibrillation or atrial fibrillation diagnosed after stroke. Int J Cardiol. 2018;261:68–72.

66. Dahlin AA, Parsons CC, Barengo NC, Ruiz JG, Ward-Peterson M, Zevallos JC. Association of ventricular arrhythmia and in-hospital mortality in stroke patients in Florida. Medicine (Baltimore). 2017;96.

67. Li S, Zhao X, Wang C, Liu L, Liu G, Wang Y, et al. Risk factors for poor outcome and mortality at 3 months after the ischemic stroke in patients with atrial fibrillation. J Stroke Cerebrovasc Dis. 2013;22.

68. Stead LG, Gilmore RM, Bellolio MF, Vaidyanathan L, Weaver AL, Decker WW, et al. Prolonged QTc as a Predictor of Mortality in Acute Ischemic Stroke. J Stroke Cerebrovasc Dis. 2009;18:469–74.

69. Abboud H, Berroir S, Labreuche J, Orjuela K, Amarenco P. Insular involvement in brain infarction increases risk for cardiac arrhythmia and death. Ann Neurol. 2006;59:691–9.

70. Colivicchi F, Bassi A, Santini M, Caltagirone C. Prognostic implications of right-sided insular damage, cardiac autonomic derangement, and arrhythmias after acute ischemic stroke. Stroke. 2005;36:1710–5.

71. Afsar N, Fak AS, Metzger JT, Van Melle G, Kappenberger L, Bogousslavsky J. Acute stroke increases QT dispersion in patients without known cardiac diseases. Arch Neurol. 2003;60:346–50.

72. Alkhachroum AM, Miller B, Chami T, Tatsuoka C, Sila C. A troponin study on patients with ischemic stroke, intracerebral hemorrhage and subarachnoid hemorrhage: Type II myocardial infarction is significantly associated with stroke severity, discharge disposition and mortality. J Clin Neurosci. 2019;64:83–8.

73. Kang K, Park TH, Kim N, Jang MU, Park SS, Park JM, et al. Recurrent Stroke, Myocardial Infarction, and Major Vascular Events during the First Year after Acute Ischemic Stroke: The Multicenter Prospective Observational Study about Recurrence and Its Determinants after Acute Ischemic Stroke i. J Stroke Cerebrovasc Dis. 2016;25:656–64.

74. Hoshino A, Nakamura T, Enomoto S, Kawahito H, Kurata H, Nakahara Y, et al. Prevalence of coronary artery disease in Japanese patients with cerebral infarction - Impact of metabolic syndrome and intracranial large artery atherosclerosis. Circ J. 2008;72:404–8.

75. Gongora-Rivera F, Labreuche J, Jaramillo A, Steg PG, Hauw JJ, Amarenco P. Autopsy prevalence of coronary atherosclerosis in patients with fatal stroke. Stroke. 2007;38:1203–10.

76. Prosser J, MacGregor L, Lees KR, Diener HC, Hacke W, Davis S. Predictors of early cardiac morbidity and mortality after ischemic stroke. Stroke. 2007;38:2295–302.

77. Dhamoon MS, Sciacca RR, Rundek T, Sacco RL, Elkind MSV. Recurrent stroke and cardiac risks after first ischemic stroke: The Northern Manhattan Study. Neurology. 2006;66:641–6.

78. Hashimoto N, Watanabe T, Tamura H, Tsuchiya H, Wanezaki M, Kato S, et al. Left atrial remodeling index is a feasible predictor of poor prognosis in patients with acute ischemic stroke. Heart Vessels. 2019;34:1936–43.

79. Li Y, Fitzgibbons TP, McManus DD, Goddeau RP, Silver B, Henninger N. Left Ventricular Ejection Fraction and Clinically Defined Heart Failure to Predict 90-Day Functional Outcome After Ischemic Stroke. J Stroke Cerebrovasc Dis. 2019;28:371–80.

80. Gasiorek P, Sakowicz A, Banach M, Von Haehling S, Bielecka-Dabrowa A. Arterial Stiffness and Indices of Left Ventricular Diastolic Dysfunction in Patients with Embolic Stroke of Undetermined Etiology. Dis Markers. 2019;2019.

81. Rojek A, Gasecki D, Fijalkowski M, Kowalczyk K, Kwarciany M, Wolf J, et al. Left ventricular ejection fraction and aortic stiffness are independent predictors of neurological outcome in acute ischemic stroke. J Hypertens. 2016;34:2441–8.

82. Olsen FJ, Jørgensen PG, Møgelvang R, Jensen JS, Fritz-Hansen T, Bech J, et al. Diastolic myocardial dysfunction by tissue Doppler imaging predicts mortality in patients with cerebral infarction. Int J Cardiovasc Imaging. 2015;31:1413–22.

83. Ntaios G, Papavasileiou V, Makaritsis K, Milionis H, Michel P, Vemmos K. Association of ischaemic stroke subtype with long-term cardiovascular events. Eur J Neurol. 2014;21:1108–14.

84. Seo JY, Lee KB, Lee J.G., Kim JS, Roh H, Ahn MY, et al. Implication of left ventricular diastolic dysfunction in cryptogenic ischemic stroke. Stroke. 2014;45:2757–61.

85. Ambrosi P, Singeorzan S, Oddoze C, Arques S, Heim M. Correlation of NT-proBNP with diastolic left ventricular function in elderly patients with ischemic stroke. Int J Cardiol. 2010;140:126–8.

86. Verschoof MA, Groot AE, Vermeij JD, Westendorp WF, van den Berg SA, Nederkoorn PJ, et al. Association Between Low Blood Pressure and Clinical Outcomes in Patients With Acute Ischemic Stroke. Stroke. 2019.

87. de Havenon A, Stoddard G, Saini M, Wong KH, Tirschwell D, Bath P. Increased blood pressure variability after acute ischemic stroke increases the risk of death: A secondary analysis of the Virtual International Stroke Trial Archive. JRSM Cardiovasc Dis. 2019;8:204800401985649.

88. Lee KJ, Kim BJ, Han MK, Kim JT, Cho KH, Shin DI, et al. Predictive value of pulse pressure in acute ischemic stroke for future major vascular events. Stroke. 2018;49:46–53.

89. Kang J, Hong JH, Jang MU, Choi NC, Lee JS, Kim BJ, et al. Change in blood pressure variability in patients with acute ischemic stroke and its effect on early neurologic outcome. PLoS One. 2017;12:e0189216.

90. Bangalore S, Schwamm L, Smith EE, Hellkamp AS, Suter RE, Xian Y, et al. Blood pressure and in-hospital outcomes in patients presenting with ischaemic stroke. Eur Heart J. 2017;38:2827–35.

91. Lee M, Ovbiagele B, Hong KS, Wu YL, Lee JE, Rao NM, et al. Effect of Blood Pressure Lowering in Early Ischemic Stroke: Meta-Analysis. Stroke. 2015;46:1883–9.

92. Zhao R, Liu FD, Wang S, Peng JL, Tao XX, Zheng B, et al. Blood Pressure Reduction in the Acute Phase of an Ischemic Stroke Does Not Improve Short- or Long-Term Dependency or Mortality: A Meta-Analysis of Current Literature. Medicine (Baltimore). 2015;94:e896.

93. Manning LS, Rothwell PM, Potter JF, Robinson TG. Prognostic Significance of Short-Term Blood Pressure Variability in Acute Stroke: Systematic Review. Stroke. 2015;46:2482–90.

94. Oh MS, Yu KH, Hong KS, Kang DW, Park JM, Bae HJ, et al. Modest blood pressure reduction with valsartan in acute ischemic stroke: A prospective, randomized, open-label, blinded-end-point trial. Int J Stroke. 2015;10:745–51.

95. He J, Zhang Y, Xu T, Zhao Q, Wang D, Chen CS, et al. Effects of immediate blood pressure reduction on death and major disability in patients with acute ischemic stroke: the CATIS randomized clinical trial. JAMA. 2014;311:479–89.

96. Sandset EC, Murray GD, Bath PMW, Kjeldsen SE, Berge E. Relation between change in blood pressure in acute stroke and risk of early adverse events and poor outcome. Stroke. 2012;43:2108–14.

97. Aries MJH, Bakker DC, Stewart RE, De Keyser J, Elting JWJ, Thien T, et al. Exaggerated postural blood pressure rise is related to a favorable outcome in patients with acute ischemic stroke. Stroke. 2012;43:92–6.

98. Geeganage C, Tracy M, England T, Sare G, Moulin T, Woimant F, et al. Relationship between baseline blood pressure parameters (including mean pressure, pulse pressure, and variability) and early outcome after stroke: Data from the tinzaparin in acute ischaemic stroke trial (TAIST). Stroke. 2011;42:491–3.

99. Tomii Y, Toyoda K, Suzuki R, Naganuma M, Fujinami J, Yokota C, et al. Effects of 24-hour blood pressure and heart rate recorded with ambulatory blood pressure monitoring on recovery from acute ischemic stroke. Stroke. 2011;42:3511–7.

100. Sandset EC, Bath PMW, Boysen G, Jatuzis D, Kõrv J, Lüders S, et al. The angiotensin-receptor blocker candesartan for treatment of acute stroke (SCAST): a randomised, placebo-controlled, double-blind trial. Lancet. 2011;377:741–50.

101. Sare GM, Ali M, Shuaib A, Bath PMW. Relationship between hyperacute blood pressure and outcome after ischemic stroke: Data from the VISTA collaboration. Stroke. 2009;40:2098–103.

102. Bath PMW, Martin RH, Palesch Y, Cotton D, Yusuf S, Sacco R, et al. Effect of telmisartan on functional outcome, recurrence, and blood pressure in patients with acute mild ischemic stroke: A PRoFESS subgroup analysis. Stroke. 2009;40:3541–6.

103. Potter JF, Robinson TG, Ford GA, Mistri A, James M, Chernova J, et al. Controlling hypertension and hypotension immediately post-stroke (CHHIPS): a randomised, placebo-controlled, double-blind pilot trial. Lancet Neurol. 2009;8:48–56.

104. Eveson DJ, Robinson TG, Potter JF. Lisinopril for the Treatment of Hypertension Within the First 24 Hours of Acute Ischemic Stroke and Follow-Up. Am J Hypertens. 2007;20:270–7.

105. Rodríguez-García JL, Botia E, De La Sierra A, Villanueva MA, González-Spínola J. Significance of elevated blood pressure and its management on the short-term outcome of patients with acute ischemic stroke. Am J Hypertens. 2005;18:379–84.

106. Aslanyan S, Fazekas F, Weir CJ, Horner S, Lees KR. Effect of blood pressure during the acute period of ischemic stroke on stroke outcome: A tertiary analysis of the GAIN International Trial. Stroke. 2003;34:2420–5.

107. Schrader J, Lüders S, Kulschewski A, Berger J, Zidek W, Treib J, et al. The ACCESS Study: evaluation of Acute Candesartan Cilexetil Therapy in Stroke Survivors. Stroke. 2003;34:1699–703.

108. Vlcek M, Schillinger M, Lang W, Lalouschek W, Bur A, Hirschl MM. Association Between Course of Blood Pressure Within the First 24 Hours and Functional Recovery After Acute Ischemic Stroke. Ann Emerg Med. 2003;42:619–26.

109. Horn J, de Haan RJ, Vermeulen M, Limburg M. Very Early Nimodipine Use in Stroke (VENUS): a randomized, double-blind, placebo-controlled trial. Stroke. 2001;32:461–5.

110. Ahmed N, Näsman P, Wahlgren NG. Effect of intravenous nimodipine on blood pressure and outcome after acute stroke. Stroke. 2000;31:1250–5.

111. Kaste M, Fogelholm R, Erilä T, Palomäki H, Murros K, Rissanen A, et al. A randomized, double-blind, placebo-controlled trial of nimodipine in acute ischemic hemispheric stroke. Stroke. 1994;25:1348–53.

112. Csecsei P, Pusch G, Ezer E, Berki T, Szapary L, Illes Z, et al. Relationship between Cardiac Troponin and Thrombo-Inflammatory Molecules in Prediction of Outcome after Acute Ischemic Stroke. J Stroke Cerebrovasc Dis. 2018;27:951–6.

113. Batal O, Jentzer J, Balaney B, Kolia N, Hickey G, Dardari Z, et al. The prognostic significance of troponin I elevation in acute ischemic stroke. J Crit Care. 2016;31:41–7.

114. Beaulieu-Boire I, Leblanc N, Berger L, Boulanger JM. Troponin elevation predicts atrial fibrillation in patients with stroke or transient ischemic attack. J Stroke Cerebrovasc Dis. 2013;22:978–83.

115. He L, Wang J, Dong W. The clinical prognostic significance of hs-cTnT elevation in patients with acute ischemic stroke. BMC Neurol. 2018;18.

116. Peddada K, Cruz-Flores S, Goldstein LB, Feen E, Kennedy KF, Heuring T, et al. Ischemic Stroke with Troponin Elevation: Patient Characteristics, Resource Utilization, and In-Hospital Outcomes. Cerebrovasc Dis. 2016;42:213–23.

117. Maoz A, Rosenberg S, Leker RR. Increased High-Sensitivity Troponin-T Levels Are Associated with Mortality After Ischemic Stroke. J Mol Neurosci. 2015;57:160–5.

118. Fure B, Bruun Wyller T, Thommessen B. Electrocardiographic and troponin T changes in acute ischaemic stroke. J Intern Med. 2006;259:592–7.
